# Machine learning for predicting mortality in women diagnosed with breast cancer in the State of Mato Grosso, Brazil using linked population-based cancer registry and mortality data

**DOI:** 10.1371/journal.pone.0356621

**Published:** 2026-09-18

**Authors:** Sancho Pedro Xavier, Audêncio Victor, Ana Raquel Ernesto Manuel Gotine, Fernando Henrique de Albuquerque Maia, Manuel Mahoche, Marco Aurélio Bertúlio das Neves, Alexandre Dias Porto Chiavegatto Filho, Noemi Dreyer Galvão, Ageo Mario Cândido da Silva

**Affiliations:** 1 School of Public Health, University of São Paulo (USP), São Paulo, São Paulo, Brazil; 2 Faculty of Epidemiology and Population Health, London School of Hygiene & Tropical Medicine, London, United Kingdom; 3 Institute of Collective Health, Federal University of Mato Grosso (UFMT), Cuiabá, Mato Grosso, Brazil; 4 State Secretary of Health of Mato Grosso (SES-MT), Cuiabá, Mato Grosso, Brazil; Sichuan University, CHINA

## Abstract

**Background:**

Breast cancer is the most frequently diagnosed malignancy and a leading cause of cancer-related mortality among women worldwide. In Brazil, pronounced regional inequalities persist in access to timely diagnosis and treatment, particularly in large and socioeconomically heterogeneous states such as Mato Grosso. This study aimed to develop and validate machine learning (ML) models to predict 1-, 3-, 5-, and 10-year all-cause and breast cancer-specific mortality among women diagnosed with breast cancer in Mato Grosso, Brazil.

**Methods:**

A retrospective, population-based cohort study was conducted, including 7,815 women diagnosed with breast cancer between 2001 and 2018. The dataset was randomly split into training (75%) and testing (25%) sets. Logistic regression, Random Forest, XGBoost, CatBoost, and LightGBM models were developed to predict all-cause and breast cancer-specific mortality at 1, 3, 5, and 10 years. Model performance was primarily evaluated using the area under the receiver operating characteristic curve (AUROC). Calibration was assessed using calibration plots and the Brier score (BS), with 95% confidence intervals estimated via bootstrap resampling. Model interpretability was evaluated using Shapley Additive Explanations (SHAP).

**Results:**

Gradient boosting models consistently demonstrated superior performance across prediction horizons. For all-cause mortality, CatBoost achieved the highest discrimination at 1 year (AUROC 83.18%, 95% CI 80.01-86.33), while LightGBM showed higher discrimination at 3 and 5 years (AUROC 81.01%, 95% CI 78.63-83.42; and AUROC 78.39%, 95% CI 75.99-80.64, respectively). For breast cancer-specific mortality, logistic regression achieved the highest AUROC at 1 year (84.44%, 95% CI 81.04-87.35), whereas LightGBM achieved the highest at 3 years (AUROC 79.82%, 95% CI 77.15-82.34), XGBoost at 5 years (AUROC 80.79%, 95% CI 78.54-83.23). SHAP analyses consistently identified age at diagnosis, metastatic stage, histological diagnosis, invasive ductal carcinoma (IDC), and marital status as the most influential predictors.

**Conclusions:**

ML models demonstrated good and stable discriminative performance in predicting breast cancer mortality using routine population-based data. These models may support the early identification of high-risk patients and help guide risk stratification and follow-up planning in resource-constrained health systems. However, the models have not yet been externally validated, and their generalizability to populations beyond Mato Grosso remains uncertain. External validation in independent populations is therefore warranted before broader implementation in other regions of Brazil.

## Introduction

Breast cancer persists as the most diagnosed malignancy and a leading cause of cancer-related mortality among women globally [1]. In 2022, global cancer statistics reported nearly 20 million new cases and 9.7 million deaths, with breast cancer accounting for a substantial proportion of this burden [1]. Projections suggest that breast cancer mortality will continue to increase through 2040, driven mainly by population growth, population ageing, and persistent inequities in access to early diagnosis and effective treatment [2]. Despite advances in screening and therapy, breast cancer outcomes remain highly heterogeneous [3]. Marked disparities persist in incidence, stage at diagnosis, treatment availability, and survival, particularly between high-income and low- and middle-income countries (LMICs) and within nations characterised by pronounced socioeconomic and geographic inequalities [1,2].

Brazil exemplifies this unequal landscape. Although the Unified Health System (SUS) provides universal health coverage in principle, breast cancer outcomes remain strongly shaped by social inequality, geographic accessibility, and discontinuities along the diagnostic and therapeutic pathway [4]. National studies consistently demonstrate substantial regional variation in breast cancer incidence, stage at diagnosis, and mortality, with less favourable trends observed outside major metropolitan centres and in regions with lower socioeconomic development [5–7].

Mato Grosso is characterised by vast territorial extension, low population density, long travel distances to specialised oncology services, and marked heterogeneity between metropolitan and rural municipalities [8]. Population-based analyses have documented increasing trends in overall cancer mortality in the state between 2000 and 2018, with breast cancer among the leading causes of cancer death among women [9]. In the state capital, Cuiabá, breast cancer incidence increased between 2008 and 2016 while mortality remained relatively stable, suggesting improvements in detection without proportional gains in survival [10]. This pattern underscores the need for effective risk stratification to better prioritize patient care. However, most established prognostic models have been developed in high-income settings with standardized healthcare systems [11].

In this context, machine learning (ML) models offer a potential approach to support early risk stratification using routinely collected registry data, particularly in settings where diagnostic staging may be delayed or incomplete. In practice, such models may assist in prioritizing patients for diagnostic completion, referral, and follow-up, as well as in informing resource allocation within the health system [12]. Their relevance is especially pronounced in regions such as Mato Grosso, where late-stage diagnosis and uneven access to treatment remain persistent challenges [13].

Building on this, ML methods offer a promising approach to address these gaps [14], as they can model non-linear relationships, complex interactions, and heterogeneous data structures using routinely collected clinical and administrative data. While clinical staging systems and established prognostic nomograms remain the gold standard for breast cancer risk assessment, ML approaches have demonstrated competitive performance in predicting survival compared with traditional regression models [15–17]. To the best of our knowledge, there is a paucity of studies in Brazil applying ML techniques to population-based cancer registry data to predict survival among women diagnosed with breast cancer [18]. In this context, this study leverages linked cancer registry and mortality data from Mato Grosso to enable risk stratification using routinely collected information in real-world settings. Therefore, this study aimed to develop and validate ML models to predict 1-, 3-, 5-, and 10-year all-cause and breast cancer-specific mortality among women diagnosed with breast cancer in Mato Grosso, Brazil.

## Methods

### Study design, data collection, and context

This retrospective cohort study used integrated data from the Population-Based Cancer Registry (RCBP) and the Mortality Information System (SIM), linked through deterministic matching based on patient name, mother's name, and date of birth (S1 Fig in S1 File) [19]. Cancer incidence information was obtained from the RCBP for the period 2001-2018, while mortality data covered the years 2001-2022. All data were provided by the State Secretary of Health of Mato Grosso (SES-MT). The characterization of the state of Mato Grosso, its geographical location, population distribution, and oncology infrastructure has been detailed in a previously published study [8,9].

### Study population and sample size

The study population included all patients diagnosed with breast cancer, coded as C50.0-C50.9 according to the International Classification of Diseases for Oncology, Third Edition (ICD-O-3) [20], between 2001 and 2018. Initially, a total of 7,953 patients were identified. Male patients (n = 68) were excluded due to their low number and distinct clinical characteristics, to ensure a more homogeneous analytical sample. Additional exclusions included patients with non-applicable staging (n = 1), missing data on age (n = 18), and missing information on diagnostic method (n = 51). Consequently, a total of 7,815 patients were included in the final analysis (see S1 Fig in S1 File). Variables coded as “Unknown” (e.g., educational level, marital status, and stage at diagnosis) were retained as distinct categories. These do not represent missing data but reflect information recorded as unavailable at the time of registration and may capture differences in data quality or access to care.

### Study variables

The outcome variable was mortality, defined as a binary indicator across multiple prediction horizons (1, 3, 5, and 10 years). Event status was coded as 1 for death (all-cause and breast cancer-specific mortality) occurring within the corresponding prediction horizon and 0 otherwise. Survival time was calculated from diagnosis to death or end of follow-up, whichever occurred first. For each prediction horizon, individuals who died within the specified time frame were classified as events, whereas individuals not meeting the event definition were classified as non-events. Predictor variables and data preprocessing procedures are detailed in Tables 1 and 2.

**Table 1:** Variables included in the ML models, category definitions, and preprocessing methods.

Feature type	Variable	Categories	Preprocessing method
Continuous	Age	Continuous variable (years)	Standardization using Z-score normalization
Categorical	Skin colour (race)	White; Black; Other/Unknown	One-hot encoding
	Marital status	Married; Not married; Unknown	
	Place of residence	Cuiabá; Várzea Grande; Rondonópolis; Sinop; Other municipalities	
	Histopathological type	Ductal carcinoma in situ (DCIS); Invasive ductal carcinoma (IDC); Lobular carcinoma in situ (LCIS); Other histological types	
	Diagnostic method	Cytology/clinical; Histology; Other	
	Educational level	No formal education; Primary education; Secondary education; Higher education; Unknown	
	Stage at diagnosis	In situ; Localized; Metastatic; Unknown	

**Table 2:** Distribution of predictors according to all-cause and breast cancer-specific mortality.

	Mortality					
Predictors	all-cause			breast cancer-specific		Log-rank
	Total (n = 7815)	Alive	Deceased	Alive	Deceased	
**Age (years)**						
≤ 39	1112 (14.2)	753 (67.72)	359 (32.28)	766 (68.88)	346 (31.12)	< 0.001
40-49	2161 (27.7)	1533 (70.94)	628 (29.06)	1571 (72.70)	590 (27.30)	
50-64	2877 (36.8)	1978 (68.75)	899 (31.25)	2041 (70.94)	836 (29.06)	
≥ 65	1665 (21.3)	949 (57.00)	716 (43.00)	1048 (62.94)	617 (37.06)	
**Skin colour (race)**						
White	2728 (34.9)	1674 (61.36)	1054 (38.64)	1753 (64.26)	975 (35.74)	< 0.001
Black	355 (4.5)	184 (51.83)	171 (48.17)	194 (54.65)	161 (45.35)	
Others/Unknown	4732 (60.6)	3355 (70.90)	1377 (29.10)	3479 (73.52)	1253 (26.48)	
**Educational level**						
No formal education	217 (2.8)	58 (26.73)	159 (73.27)	65 (29.95)	152 (70.05)	< 0.001
Primary education	1484 (19.0)	635 (42.79)	849 (57.21)	686 (46.23)	798 (53.77)	
Secondary education	755 (9.7)	343 (45.43)	412 (54.57)	357 (47.28)	398 (52.72)	
Higher education	679 (8.7)	388 (57.14)	291 (42.86)	398 (58.62)	281 (41.38)	
Unknown	4680 (59.9)	3789 (80.96)	891 (19.04)	3920 (83.76)	760 (16.24)	
**Marital status**						
Married	2082 (26.6)	1097 (52.69)	985 (47.31)	1160 (55.72)	922 (44.28)	< 0.001
Not married	2055 (26.3)	986 (47.98)	1069 (52.02)	1072 (52.17)	983 (47.83)	
Unknown	3678 (47.1)	3130 (85.10)	548 (14.90)	3194 (86.84)	484 (13.16)	
**Place of residence**						
Cuiabá	2788 (35.7)	1972 (70.73)	816 (29.27)	2052 (73.60)	736 (26.40)	< 0.001
Várzea Grande	718 (9.2)	477 (66.43)	241 (33.57)	498 (69.36)	220 (30.64)	
Rondonópolis	674 (8.6)	458 (67.95)	216 (32.05)	477 (70.77)	197 (29.23)	
Sinop	294 (3.8)	201 (68.37)	93 (31.63)	203 (69.05)	91 (30.95)	
Other municipalities	3341 (42.8)	2105 (63.01)	1236 (36.99)	2196 (65.73)	1145 (34.27)	
**Diagnostic method**						
Cytology/clinical	225 (2.9)	162 (72.00)	63 (28.00)	169 (75.11)	56 (24.89)	< 0.001
Histology	7043 (90.1)	5028 (71.39)	2015 (28.61)	5226 (74.20)	1817 (25.80)	
Other	547 (7.0)	23 (4.20)	524 (95.80)	31 (5.67)	516 (94.33)	
**Histopathological type**						
DCIS	231 (3.00)	212 (91.77)	19 (8.23)	214 (92.64)	17 (7.36)	< 0.001
IDC	5625 (72.0)	3956 (70.33)	1669 (29.67)	4108 (73.03)	1517 (26.97)	
LCIS	28 (0.4)	24 (85.71)	4 (14.29)	24 (85.71)	4 (14.29)	
Other	1931 (24.7)	1021 (52.87)	910 (47.13)	1080 (55.93)	851 (44.07)	
**Stage at diagnosis**						
In Situ	300 (3.8)	268 (89.33)	32 (10.67)	271 (90.33)	29 (9.67)	< 0.001
Localized	3014 (38.6)	2307 (76.54)	707 (23.46)	2368 (78.57)	646 (21.43)	
Metastatic	1311 (16.8)	734 (55.99)	577 (44.01)	778 (59.34)	533 (40.66)	
Unknown	3190 (40.8)	1904 (59.69)	1286 (40.31)	2009 (62.98)	1181 (37.02)	
**Year of diagnosis**						
≤2005	1599 (20.5)	991 (61.98)	608 (38.02)	1028 (64.29)	571 (35.71)	< 0.001
2006-2011	2241 (28.7)	1236 (55.15)	1005 (44.85)	1337 (59.66)	904 (40.34)	
2012-2018	3975 (50.9)	2986 (75.12)	989 (24.88)	3061 (77.01)	914 (22.99)	

### Data preprocessing

Several preprocessing techniques were applied to create a suitable environment for model development (Table 1). Categorical variables were transformed using one-hot encoding, generating dummy variables for each category, while numerical variables were normalized using Z-score.

### Model development and performance

Various ML algorithms were trained, including Logistic Regression (LR), Random Forest (RF), XGBoost [21], CatBoost [22], and LightGBM [23]. These models were selected due to their widespread use in health classification problems [23]. The analyses were conducted in Python (v3.12.4), using the core libraries pandas (2.2.2), NumPy (1.26.4), and scikit-learn (1.4.2). The official libraries for CatBoost (1.2.7), XGBoost (2.1.3), and LightGBM (4.5.0) were employed for training their respective models. For specific hyperparameters, the search ranges were defined based on existing literature [24,25].

The tested hyperparameters and those selected as optimal are presented in the supplementary material (S4 Table in S1 File). Hyperparameter optimization was performed using GridSearchCV with stratified five-fold cross-validation (random_state = 42) to preserve the outcome distribution across folds [25,26]. The dataset was split into training and test sets using the *train_test_split* function, with 25% of the sample reserved for testing. Stratified sampling was applied to ensure that the proportion of the mortality outcome was maintained in both datasets. No resampling or class balancing techniques were applied to address outcome imbalance, in order to preserve the original distribution of events.

### Model evaluation, validation and interpretation

Model performance was evaluated on the test set using accuracy, precision, sensitivity, specificity, F1-score, Matthews Correlation Coefficient (MCC), and the area under the ROC curve (AUROC), which was considered the primary metric for model comparison. Differences between the ROC curves of the models were assessed using the DeLong test [27], while 95% confidence intervals (95% CI) for the AUC were obtained using the bootstrap method with 2,000 repetitions.

Model robustness and generalizability were evaluated for the highest-performing model using an envelope of ROC curves generated by perturbing the standardized age variable in the test set with Gaussian noise (σ = 0.01, 0.05, and 0.1) across 500 simulations, alongside the ROC curve from the original test data.

The optimal classification threshold was determined using Youden's index, defined as the point on the ROC curve that maximizes the sum of sensitivity and specificity [25]. To ensure comparability between models regarding sensitivity and specificity, the optimal threshold obtained from the superior model at each time horizon was subsequently applied to the other models. McNemar's test, a non-parametric test for paired data, was used to compare the accuracy, sensitivity, and specificity between two models, considering the best threshold determined by Youden's index [27].

The Brier Score was also calculated to quantitatively assess both discrimination and calibration [28], according to the following formula:


$$\begin{array}{c} (Y,P)=\frac{\text{1}}{n}*\sum_{i=\text{1}}^{n}{\left(P_{i}-Y_{i}\right)}^{\text{2}} \end{array}$$

where $P_{i}$ represents the probability predicted by the model, $Y_{i}$ the actual occurrence of the event, and n the number of observations.

This process was repeated 2,000 times via bootstrap, so that each sample was used to validate the predictions. The final Brier Score was obtained as the mean of the 2,000 iterations, accompanied by the respective 95% CI. Lower values indicate better discrimination and calibration [29]. Subsequently, calibration plots were used to assess the consistency between the models’ discriminative performance and calibration. Differences in the Brier Score between models were evaluated using the paired bootstrap test procedure with 10,000 iterations. For each time horizon, the best model was subsequently analyzed for explainability using Shapley Additive Explanations (SHAP) [30]. Positive SHAP values indicate an increased risk of mortality, while negative values suggest a reduced risk. The mean impact of variables was also assessed, highlighting their relative importance in prediction [31]. A schematic overview of the full analytical workflow is provided in S2 Fig in S1 File.

Decision curve analysis (DCA) was performed to evaluate the potential clinical utility of each model across a range of threshold probabilities [32]. Net benefit was calculated for each model and compared with the “treat all” and “treat none” strategies. Models with higher net benefit at a given threshold probability were considered to provide greater potential clinical utility.

### Statistical analysis

Descriptive analyses of categorical variables were performed using absolute and relative frequencies. Differences in survival between groups were assessed using the log-rank test [33].

### Ethics approval and consent to participate

This study was conducted in accordance with the Declaration of Helsinki and approved by the Institutional Research Ethics Committee (approval number: 4.858.521). Secondary data were obtained from the RCBP and the SIM, with authorization for research use granted on 20 July 2021. Identifiable information was temporarily accessed solely for the purpose of deterministic record linkage between databases. After completion of the linkage process, all personal identifiers were removed, and analyses were conducted using fully anonymised data. The requirement for informed consent was waived due to the retrospective nature of the study and the use of secondary data.

## Results

### Sociodemographic and clinical characteristics

A total of 7,815 women diagnosed with breast cancer between 2001 and 2018 were included in the analysis. Table 2 presents the distribution of sociodemographic and clinical characteristics according to all-cause and breast cancer-specific mortality, with statistically significant differences observed for all evaluated predictors (log-rank test, p < 0.001). All-cause mortality increased with age, reaching 43.0% among women aged ≥65 years, compared with 29.1-32.3% among those younger than 65 years (log-rank p < 0.001). Similar patterns were observed for breast cancer-specific mortality. Black women exhibited higher all-cause mortality (48.2%) compared with White women (38.6%; p < 0.001). Mortality was substantially higher among women with no formal education (73.3%) than among those with higher education (42.9%; p < 0.001).

Geographic disparities were evident, with lower all-cause mortality among residents of Cuiabá (29.3%) compared to other municipalities (36.9%, p < 0.001). Advanced clinical features significantly influenced survival, with metastatic disease at diagnosis associated with the highest mortality (44.0% all-cause; 40.7% breast cancer-specific), while in situ disease showed the most favourable outcomes with 10% mortality (p < 0.001). Survival improved over time, as seen in lower mortality among women diagnosed between 2012 and 2018 (24.9%) compared to those diagnosed before 2006 (38.0%).

### Model performance for all-cause mortality

**Fig 1:**
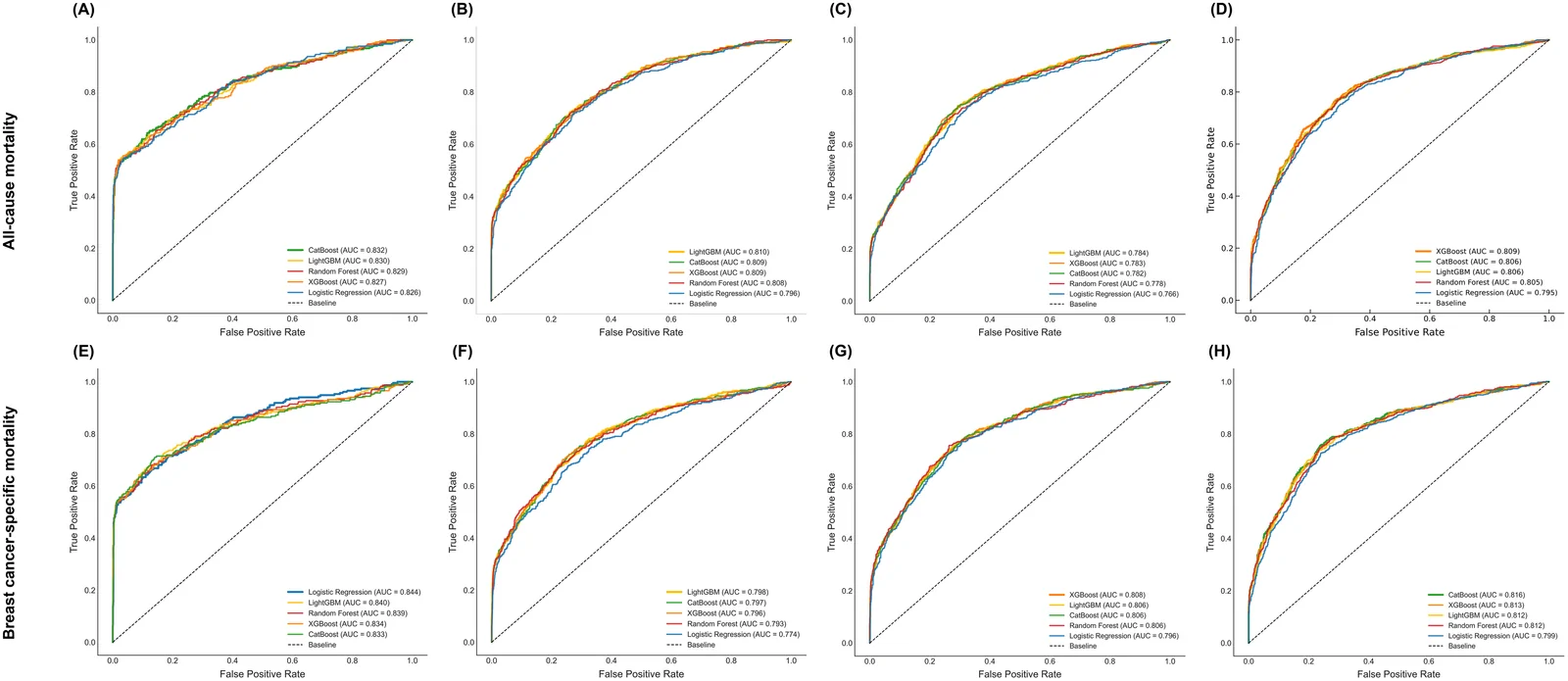
ROC curves for all-cause and breast cancer-specific mortality. Panels A-D show predictions of all-cause mortality at 1, 3, 5, and 10 years, respectively, while panels E-H show predictions of breast cancer-specific mortality at the same time horizons.

**Fig 2:**
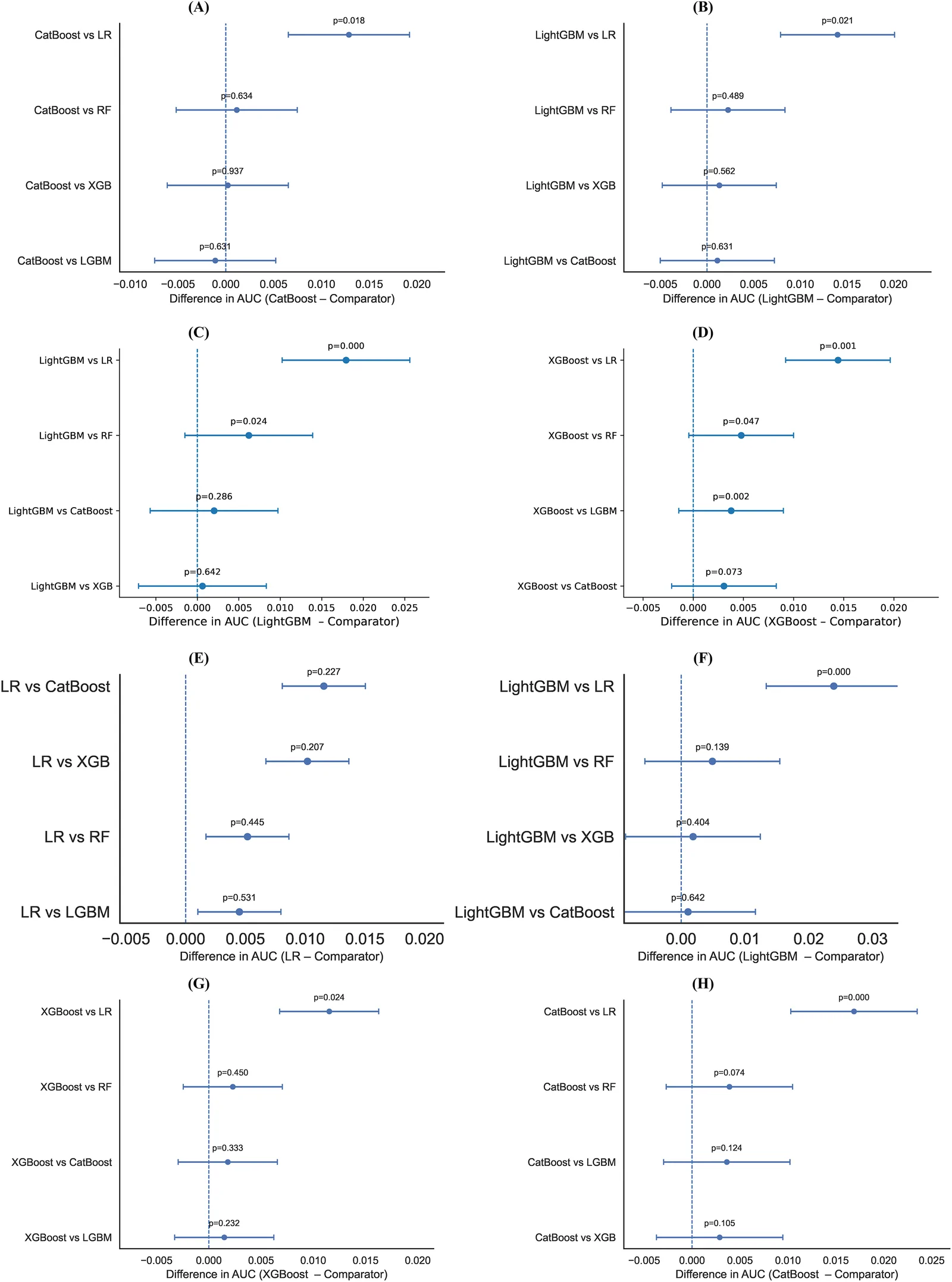
DeLong test results comparing the discriminative performance of the model with the highest AUC-ROC against the remaining models across prediction time horizons. The model with the highest AUC-ROC was used as the reference and sequentially compared with the others. Panels A-D present comparisons for all-cause mortality at 1, 3, 5, and 10 years, respectively, while panels E-H show the corresponding comparisons for breast cancer-specific mortality at the same time horizons.

Predictive performance for all-cause mortality at 1-, 3-, 5-, and 10-year horizons is summarised in Table 3. Gradient boosting-based methods (CatBoost, XGBoost, and LightGBM) consistently demonstrated higher AUROC values than logistic regression and Random Forest, with statistically significant differences observed for logistic regression (DeLong test, p < 0.05) (Fig 2). ROC curves are presented in Fig 1, with robustness analyses under Gaussian noise shown in S4 Fig in S1 File.

**Table 3:** Performance of ML models for predicting all-cause mortality across time horizons (1, 3, 5, and 10 years).

Metrics	Models				
	Logistic	Random Forest	CatBoost	XGBoost	LightGBM
**1-year**				
Accuracy	91.97 (90.74-93.09)	92.32 (91.10-93.45)	92.53 (91.35-93.65)	92.48 (91.30-93.55)	92.22 (91.04-93.35)
Precision	80.65 (74.51-86.67)	89.84 (84.55-94.69)	88.41 (82.96-93.43)	87.23 (81.94-92.65)	85.71 (80.15-91.31)
Sensitivity	49.60 (43.75-55.79)	45.63 (39.36-52.00)	48.41 (42.24-54.48)	48.81 (42.64-54.79)	47.62 (41.39-53.57)
Specificity	98.00 (97.60-98.83)	99.00 (98.80-99.59)	99.06 (98.58-99.47)	99.00 (98.45-99.41)	99.00 (98.31-99.30)
F1-Score	61.43 (55.83-66.67)	60.53 (54.45-66.49)	62.56 (56.84-68.16)	62.60 (56.91-67.98)	61.22 (55.47-66.67)
MCC	59.36 (53.91- 64.76)	60.78 (55.46-66.20)	62.10 (56.80-67.41)	61.85 (56.54-67.00)	60.36 (54.73-65.67)
AUROC	82.58 (79.35-85.65)	82.95 (79.72-85.95)	**83.18** (80.01-86.33)	82.70 (79.52-85.71)	82.95 (79.84-85.99)
AUC-PR	69.96 (59.27-69.96)	65.70 (60.39-70.94)	65.91 (60.52-71.16)	65.70 (60.35-70.94)	66.01 (60.68-71.13)
Brier Score	0.069 (0.060-0.078)	0.067 (0.058-0.076)	**0.066** (0.058-0.075)	0.066 (0.057-0.076)	0.066 (0.058-0.075)
Confusion matrix	TP: 125, FP: 30, TN: 1672, FN: 127	TP: 115, FP: 13, TN: 1689, FN: 137	TP: 122, FP: 16, TN: 1686, FN: 130	TP: 123, FP: 18, TN: 1684, FN: 129	TP: 120, FP: 20, TN: 1682, FN: 132
**3-year**					
Accuracy	83.47 (81.73-85.11)	84.19 (82.55-85.72)	84.08 (82.40-85.62)	83.98 (82.24-85.57)	84.03 (82.34-85.57)
Precision	80.95 (75.37-86.46)	91.19 (86.55-95.15)	87.72 (82.72-92.40)	85.08 (79.77-90.31)	86.36 (81.14-91.28)
Sensitivity	34.77 (30.44-39.28)	32.95 (28.64-37.33)	34.09 (29.74-38.46)	35.00 (30.59-39.61)	34.55 (30.17-39.12)
Specificity	98.00 (96.80-98.37)	99.00 (98.58-99.53)	98.61 (98.01-99.15)	98.00 (97.53-98.89)	98.00 (97.75-99.01)
F1-Score	48.65 (43.82-53.55)	48.41 (43.43-53.21)	49.10 (44.26-53.87)	49.60 (44.77-54.58)	49.35 (44.48-54.22)
MCC	45.78 (41.05-50.58)	48.93 (44.53-53.16)	48.27 (43.87-52.75)	47.86 (43.27-52.68)	48.09 (43.49-52.65)
AUROC	79.61 (77.21-81.99)	80.79 (78.38-83.14)	80.90 (78.50-83.28)	80.88 (78.50-83.29)	**81.01** (78.63-83.42)
AUC-PR	62.92 (58.69-67.06)	65.51 (61.64-69.35)	65.68 (61.81-69.56)	65.84 (61.98-69.78)	66.08 (62.26-69.93)
Brier Score	0.127 (0.118-0.137)	0.123 (0.114-0.133)	0.121 (0.112-0.131)	0.121 (0.112-0.131)	**0.121** (0.112-0.131)
Confusion matrix	TP: 153, FP: 36, TN: 1478, FN: 287	TP: 145, FP: 14, TN: 1500, FN: 295	TP: 150, FP: 21, TN: 1493, FN: 290	TP: 154, FP: 27, TN: 1487, FN: 286	TP: 152, FP: 24, TN: 1490, FN: 288
**5-year**					
Accuracy	77.84 (76.00-79.73)	77.58 (75.79-79.48)	77.79 (75.95-79.58)	77.43 (75.59-79.22)	77.69 (75.74-79.48)
Precision	69.09 (63.70-74.45)	73.58 (67.73-79.71)	68.84 (63.08-74.37)	67.02 (61.23-72.54)	67.47 (61.90-72.99)
Sensitivity	35.32 (31.44-39.24)	29.00 (25.23-32.60)	35.32 (31.26-39.09)	35.50 (31.42-39.43)	36.62 (32.51-40.45)
Specificity	94.00 (92.71-95.19)	96.00 (95.03-97.07)	93.93 (92.68-95.23)	93.00 (92.02-94.66)	93.00 (91.93-94.56)
F1-Score	46.74 (42.46-50.71)	41.60 (37.17-45.75)	46.68 (42.22-50.71)	46.42 (41.93-50.47)	47.47 (43.05-51.52)
MCC	37.65 (32.99-42.29)	35.97 (31.27-40.46)	37.50 (32.78-42.08)	36.53 (31.66-41.16)	37.47 (32.51-42.11)
AUROC	76.60 (74.12-78.99)	77.77 (75.38-80.05)	78.19 (75.80-80.49)	78.33 (75.94-80.56)	**78.39** (75.99-80.64)
AUC-PR	62.29 (58.19-66.18)	63.45 (59.47-67.20)	64.44 (60.57-68.14)	64.26 (60.38-67.92)	64.41 (60.49-68.16)
Brier Score	0.156 (0.146-0.165)	0.152 (0.143-0.161)	0.151 (0.141-0.160)	0.151 (0.141-0.160)	**0.151** (0.141-0.160)
Confusion matrix	TP: 190, FP: 85, TN: 1331, FN: 348	TP: 156, FP: 56, TN: 1360, FN: 382	TP: 190, FP: 86, TN: 1330, FN: 348	TP: 191, FP: 94, TN: 1322, FN: 347	TP: 197, FP: 95, TN: 1321, FN: 341
**10-year**					
Accuracy	75.90 (74.05-77.74)	76.51 (74.67-78.45)	76.66 (74.89-78.61)	76.87 (75.03-78.81)	76.25 (74.41-78.25)
Precision	67.42 (63.16-71.46)	73.31 (68.61-77.69)	71.18 (66.67-75.46)	71.15 (66.75-75.35)	70.59 (65.95-74.93)
Sensitivity	47.68 (43.79-51.41)	41.76 (37.99-45.56)	45.44 (41.76-49.28)	46.56 (42.69-50.38)	44.16 (40.35-48.06)
Specificity	89.16 (87.49-90.72)	92.85 (91.41-94.15)	91.35 (89.81-92.92)	91.12 (89.57-92.55)	91.35 (89.78-92.80)
F1-Score	55.86 (52.31-59.27)	53.21 (49.33-56.96)	55.47 (51.88-59.22)	56.29 (52.69-59.85)	54.33 (50.69-58.02)
MCC	41.08 (36.83-45.45)	41.82 (37.37-46.21)	42.56 (38.18-47.12)	43.20 (38.86-47.74)	41.39 (37.03-45.75)
AUROC	79.49 (77.39-81.75)	80.46 (78.29-82.55)	80.63 (78.53-82.68)	**80.93** (78.86-83.04)	80.56 (78.44-82.64)
AUC-PR	67.66 (64.19-71.17)	69.95 (66.53-73.22)	69.84 (66.34-73.18)	70.68 (67.26-73.95)	70.07 (66.63-73.35)
Brier Score	0.162 (0.153-0.171)	0.159 (0.150-0.167)	0.157 (0.148-0.166)	**0.155** (0.146-0.164)	0.157 (0.147-0.166)
Confusion matrix	TP: 298, FP: 144, TN: 1185, FN: 327	TP: 261, FP: 95, TN: 1234, FN: 364	TP: 284, FP: 115, TN: 1214, FN: 341	TP: 291, FP: 118, TN: 1211, FN: 334	TP: 276, FP: 115, TN: 1214, FN: 349

Note: Values in parentheses represent 95% confidence intervals estimated using bootstrap resampling (2,000 repetitions).

At the 1-year horizon, CatBoost achieved the highest discrimination (AUROC = 83.18%; 95% CI: 80.01-86.33). At longer time horizons, AUROC values declined gradually but remained within a range from 79.61-81.01% at 3 years and from 76.60-78.39% at 5 years, with LightGBM demonstrating strong discriminative performance at both time points. At 10 years, AUROC values ranged from 79.49-80.93%, with XGBoost showing high discriminative ability.

Using a fixed probability threshold of 0.50, sensitivity ranged from 45.6% to 49.6%, while specificity remained high, ranging from 98.0% to 99.1%. After applying thresholds derived from Youden’s index, sensitivity increased substantially across all time horizons, ranging from 61.1% to 64.3% at 1 year, from 69.3% to 74.1% at 3 years, and from 75.0% to 78.6% at 10 years, as detailed in Table 4.

**Table 4:** Performance of ML models for predicting all-cause mortality at 1, 3, 5, and 10-year horizons using optimal decision thresholds defined by Youden’s index.

Metrics	Models				
	Logistic	Random Forest	CatBoost	XGBoost	LightGBM
**1-year**				
Accuracy	81.42 (79.68-83.11)	83.06 (81.42-84.70)	85.36 (83.83-86.95)	84.70 (83.16-86.34)	85.52 (83.98-87.05)
Precision	37.12 (32.75-41.80)	40.15 (35.64-45.11)	45.25 (40.42-50.56)	43.38 (38.37-48.71)	45.45 (40.34-50.89)
Sensitivity	63.49 (57.32-69.20)	63.89 (58.12-69.80)	64.29 (58.80-70.19)	61.11 (55.09-67.07)	61.51 (55.51-67.51)
Specificity	84.08 (82.33-85.82)	85.90 (84.24-87.59)	88.48 (86.99-89.98)	88.19 (86.62-89.71)	89.07 (87.54-90.48)
F1-Score	46.85 (42.18-51.31)	49.31 (44.78-53.98)	53.11 (48.59-57.92)	50.74 (45.99-55.81)	52.28 (47.49-57.15)
MCC	38.45 (33.33-43.56)	41.32 (36.27-46.52)	45.72 (40.74-51.00)	42.86 (37.60-48.33)	44.66 (39.43-49.97)
AUC-PR	64.79 (59.36-70.00)	65.73 (60.46-70.99)	65.94 (60.59-71.21)	65.72 (60.40-70.98)	66.11 (60.74-71.18)
Confusion matrix	TP: 160, FP: 271, TN: 1431, FN: 92	TP: 161, FP: 240, TN: 1462, FN: 91	TP: 162, FP: 196, TN: 1506, FN: 90	TP: 154, FP: 201, TN: 1501, FN: 98	TP: 155, FP: 186, TN: 1516, FN: 97
**3-year**					
Accuracy	72.52 (70.57-74.52)	71.08 (69.19-73.13)	72.47 (70.62-74.51)	73.34 (71.44-75.28)	73.44 (71.55-75.38)
Precision	43.25 (39.78-46.96)	41.96 (38.70-45.48)	43.29 (39.94-47.00)	44.14 (40.66-47.87)	44.43 (40.92-48.11)
Sensitivity	70.68 (66.38-74.89)	74.09 (70.11-78.17)	71.82 (67.49-76.08)	69.32 (64.94-73.56)	71.59 (67.25-75.89)
Specificity	73.05 (70.99-75.28)	70.21 (68.04-72.52)	72.66 (70.52-74.82)	74.50 (72.40-76.62)	73.98 (71.74-76.10)
F1-Score	53.67 (50.13-57.12)	53.57 (50.29-56.96)	54.02 (50.74-57.57)	53.93 (50.46-57.43)	54.83 (51.50-58.27)
MCC	37.88 (33.68-42.04)	37.81 (33.77-41.97)	38.40 (34.35-42.82)	38.29 (33.99-42.69)	39.59 (35.50-44.09)
AUC-PR	62.96 (58.80-67.11)	65.53 (61.70-69.40)	65.70 (61.87-69.60)	65.87 (62.05-69.82)	66.07 (62.32-69.97)
Confusion matrix	TP: 311, FP: 408, TN: 1106, FN: 129	TP: 326, FP: 451, TN: 1063, FN: 114	TP: 316, FP: 414, TN: 1100, FN: 124	TP: 305, FP: 386, TN: 1128, FN: 135	TP: 315, FP: 394, TN: 1120, FN: 125
**5-year**					
Accuracy	69.50 (67.40-71.39)	69.14 (66.99-71.03)	71.55 (69.55-73.49)	71.19 (69.14-73.03)	71.75 (69.81-73.69)
Precision	46.56 (43.06-49.88)	46.26 (42.79-49.48)	48.91 (45.43-52.33)	48.49 (44.99-51.90)	49.14 (45.56-52.66)
Sensitivity	72.86 (69.14-76.58)	74.72 (71.15-78.30)	74.72 (70.99-78.23)	74.72 (71.08-78.29)	74.54 (70.82-78.29)
Specificity	68.22 (65.62-70.54)	67.02 (64.47-69.34)	70.34 (67.88-72.66)	69.84 (67.35-72.08)	70.69 (68.30-72.91)
F1-Score	56.81 (53.58-59.75)	57.14 (53.81-60.14)	59.12 (55.88-62.17)	58.81 (55.55-61.86)	59.23 (55.96-62.22)
MCC	37.06 (33.00-40.99)	37.52 (33.38-41.32)	40.77 (36.57-44.69)	40.28 (36.12-44.17)	40.96 (36.93-44.85)
AUC-PR	62.32 (58.27-66.24)	63.48 (59.55-67.25)	64.47 (60.63-68.19)	64.29 (60.45-67.97)	64.45 (60.56-68.22)
Confusion matrix	TP: 392, FP: 450, TN: 966, FN: 146	TP: 402, FP: 467, TN: 949, FN: 136	TP: 402, FP: 420, TN: 996, FN: 136	TP: 402, FP: 427, TN: 989, FN: 136	TP: 401, FP: 415, TN: 1001, FN: 137
			**10-year**		
Accuracy	70.32 (68.27-72.36)	72.26 (70.27-74.21)	72.77 (70.73-74.67)	73.29 (71.34-75.18)	72.52 (70.52-74.46)
Precision	52.40 (49.10-55.51)	54.68 (51.27-57.90)	55.50 (52.19-58.76)	56.01 (52.63-59.13)	55.07 (51.62-58.25)
Sensitivity	78.56 (75.36-81.70)	77.60 (74.30-80.74)	75.04 (71.50-78.40)	76.80 (73.45-80.19)	76.48 (73.11-79.67)
Specificity	66.44 (63.87-69.06)	69.75 (67.17-72.28)	71.71 (69.26-74.16)	71.63 (69.18-74.10)	70.65 (68.19-73.15)
F1-Score	62.87 (59.95-65.47)	64.15 (61.22-66.80)	63.81 (60.78-66.45)	64.78 (61.88-67.40)	64.03 (61.23-66.62)
MCC	42.01 (38.09-45.93)	44.36 (40.49-48.13)	44.01 (39.85-47.77)	45.52 (41.56-49.25)	44.25 (40.31-47.93)
AUC-PR	67.69 (64.27-71.23)	69.97 (66.58-73.27)	69.87 (66.40-73.23)	70.70 (67.32-73.99)	70.08 (66.68-73.39)
Confusion matrix	TP: 491, FP: 446, TN: 883, FN: 134	TP: 485, FP: 402, TN: 927, FN: 140	TP: 469, FP: 376, TN: 953, FN: 156	TP: 480, FP: 377, TN: 952, FN: 145	TP: 478, FP: 390, TN: 939, FN: 147

**Note:** Values in parentheses represent 95% confidence intervals estimated using bootstrap resampling. The optimal decision threshold was defined using Youden’s index (J = 0.122) based on the superior-performing model at the 1-year time horizon and subsequently applied to all models to ensure comparability. For the 3-year prediction, Youden’s index was J = 0.224. For the 5-year and 10-year predictions, Youden’s index values were J = 0.271 and J = 0.270, respectively.

### Breast cancer-specific mortality prediction

Model performance for breast cancer-specific mortality is presented in Table 5. At the 1-year horizon, Logistic Regression demonstrated high discriminative performance (AUROC = 84.44%; 95% CI: 81.04-87.35), whereas LightGBM achieved an AUROC of 83.99% (95% CI: 80.23-87.26). Across longer time horizons, AUROC values remained relatively stable, ranging from 77.43% to 81.57%. Gradient boosting models generally showed good discriminative performance (DeLong test, p < 0.05) (Fig 2). ROC curves for the 3-, 5-, and 10-year horizons are presented in Fig 1, while ROC curves under Gaussian noise perturbation are shown in S4 Fig in S1 File. Specificity was consistently high (≥99%), while sensitivity ranged from 49.8% to 51.9% using a fixed probability threshold of 0.50.

**Table 5:** Performance of ML models for predicting breast cancer-specific mortality at 1, 3, 5, and 10-year horizons.

Metrics	Models				
	Logistic	Random Forest	CatBoost	XGBoost	LightGBM
**1-year**				
Accuracy	93.04 (91.86-94.11)	93.24 (92.12-94.37)	93.35 (92.22-94.42)	93.19 (92.07-94.27)	93.30 (92.17-94.37)
Precision	84.14 (77.78-89.66)	89.31 (83.76-94.12)	88.89 (83.33-93.75)	88.64 (82.95-93.59)	88.81 (83.21-93.70)
Sensitivity	51.91 (45.45-58.33)	49.79 (43.35-56.49)	51.06 (44.63-57.56)	49.79 (43.39-56.37)	50.64 (44.20-57.14)
Specificity	99.00 (98.12-99.18)	99.00 (98.77-99.59)	99.13 (98.67-99.54)	99.00 (98.67-99.54)	99.00 (98.67-99.53)
F1-Score	64.21 (58.35-69.63)	63.93 (57.82-69.66)	64.86 (59.05-70.18)	63.76 (57.65-69.31)	64.50 (58.49-70.11)
MCC	62.77 (56.96-68.16)	63.69 (58.12-69.12)	64.37 (58.90-69.56)	63.39 (57.75-68.74)	64.05 (58.37-69.37)
AUROC	**84.44** (81.04-87.35)	83.92 (80.26-87.09)	83.28 (79.34-86.63)	83.42 (79.61-86.69)	83.99 (80.23-87.26)
AUC-PR	65.64 (59.34-71.05)	66.38 (60.35-71.79)	66.67 (60.63-72.30)	66.33 (60.38-71.80)	66.43 (59.95-72.29)
Brier Score	0.062 (0.053-0.071)	0.060 (0.052-0.069)	0.059 (0.051-0.068)	0.060 (0.051-0.069)	**0.060** (0.050-0.068)
Confusion matrix	TP: 122, FP: 23, TN: 1696, FN: 113	TP: 117, FP: 14, TN: 1705, FN: 118	TP: 120, FP: 15, TN: 1704, FN: 115	TP: 117, FP: 15, TN: 1704, FN: 118	TP: 119, FP: 15, TN: 1704, FN: 116
**3-year**					
Accuracy	83.93 (82.34-85.62)	84.70 (83.11-86.39)	84.44 (82.86-86.13)	84.54 (82.96-86.18)	84.60 (83.01-86.28)
Precision	76.74 (70.29-82.91)	87.41 (81.63-92.59)	81.10 (75.00-86.71)	83.77 (77.70-89.24)	83.02 (77.21-88.68)
Sensitivity	32.51 (28.05-37.31)	30.79 (26.34-35.58)	32.76 (28.25-37.41)	31.77 (27.27-36.55)	32.51 (27.91-37.37)
Specificity	97.00 (96.61-98.18)	98.84 (98.26-99.35)	98.00 (97.25-98.65)	98.39 (97.71-98.97)	98.26 (97.58-98.90)
F1-Score	45.67 (40.55-50.62)	45.54 (40.29-50.88)	46.67 (41.52-51.73)	46.07 (40.88-51.28)	46.73 (41.58-51.93)
MCC	42.86 (37.72-47.85)	46.15 (41.39-50.90)	45.67 (40.18-49.93)	45.41 (40.48-50.26)	45.66 (40.67-50.57)
AUROC	77.43 (74.59-80.05)	79.33 (76.59-81.97)	79.71 (76.94-82.32)	79.63 (76.92-82.21)	**79.82** (77.15-82.34)
AUC-PR	57.97 (53.25-62.66)	61.53 (56.94-66.03)	61.54 (57.01-65.88)	61.38 (56.82-65.78)	61.71 (57.10-66.05)
Brier Score	0.126 (0.115-0.136)	0.120 (0.110-0.130)	0.119 (0.109-0.129)	0.119 (0.109-0.129)	**0.119** (0.109-0.129)
Confusion matrix	TP: 132, FP: 40, TN: 1508, FN: 274	TP: 125, FP: 18, TN: 1530, FN: 281	TP: 133, FP: 31, TN: 1517, FN: 273	TP: 129, FP: 25, TN: 1523, FN: 277	TP: 132, FP: 27, TN: 1521, FN: 274
**5-year**					
Accuracy	80.60 (78.86-82.29)	81.17 (79.38-82.91)	81.12 (79.32-82.91)	80.91 (79.07-82.70)	81.37 (79.58-83.11)
Precision	76.11 (70.71-81.69)	85.64 (80.49-90.53)	75.00 (69.53-80.26)	73.48 (68.05-78.73)	76.49 (71.31-81.78)
Sensitivity	34.61 (30.50-38.89)	31.19 (27.29-35.14)	38.63 (34.58-42.77)	39.03 (34.81-43.14)	38.63 (34.58-42.71)
Specificity	96.29 (95.35-97.24)	98.22 (97.51-98.84)	95.61 (94.51-96.61)	95.20 (94.04-96.25)	95.95 (94.92-96.92)
F1-Score	47.58 (43.08-51.93)	45.72 (41.21-50.14)	51.00 (46.77-55.04)	50.99 (46.69-55.01)	51.34 (47.02-55.43)
MCC	42.08 (37.40-46.65)	44.17 (39.75-48.41)	44.19 (39.57-48.83)	43.61 (38.92-48.18)	45.01 (40.34-49.55)
AUROC	79.64 (77.27-82.06)	80.56 (78.29-82.99)	80.61 (78.32-83.07)	**80.79** (78.54-83.23)	80.64 (78.38-83.13)
AUC-PR	63.50 (59.42-67.63)	66.76 (62.97-70.48)	66.12 (62.32-69.83)	66.10 (62.25-69.95)	65.88 (61.91-69.79)
Brier Score	0.141 (0.130-0.150)	0.136 (0.127-0.145)	0.136 (0.126-.0146)	**0.136** (0.126-0.146)	0.136 (0.12.6-0.146)
Confusion matrix	TP: 172, FP: 54, TN: 1403, FN: 325	TP: 155, FP: 26, TN: 1431, FN: 342	TP: 192, FP: 64, TN: 1393, FN: 305	TP: 194, FP: 70, TN: 1387, FN: 303	TP: 192, FP: 59, TN: 1398, FN: 305
			**10-year**		
Accuracy	77.64 (75.84-79.48)	77.94 (76.10-79.84)	78.76 (76.97-80.71)	78.45 (76.61-80.35)	78.05 (76.20-79.94)
Precision	70.57 (65.60-75.51)	76.00 (70.92-80.92)	73.04 (68.33-77.59)	71.19 (66.47-75.59)	69.23 (64.27-73.59)
Sensitivity	40.94 (36.93-45.00)	36.41 (32.54-40.55)	43.90 (39.72-48.03)	44.77 (40.54-48.89)	45.47 (41.29-49.71)
Specificity	92.90 (91.62-94.16)	95.22 (94.10-96.32)	93.26 (91.99-94.52)	92.46 (91.01-93.79)	91.59 (90.10-92.95)
F1-Score	51.82 (47.71-55.79)	49.23 (44.97-53.24)	54.84 (50.50-58.78)	54.97 (39.15-48.18)	54.89 (50.79-58.81)
MCC	40.99 (36.36-45.36)	41.43 (36.80-46.08)	44.39 (39.69-49.07)	43.70 (39.15-48.18)	42.78 (37.94-47.45)
AUROC	79.88 (77.72-82.13)	81.18 (79.05-83.37)	**81.57** (79.46-83.75)	81.28 (79.16-83.48)	81.21 (79.05-83.38)
AUC-PR	66.78 (62.73-70.45)	69.58 (65.91-73.08)	70.06 (66.31-73.48)	69.79 (66.08-73.24)	69.51 (65.78-72.90)
Brier Score	0.154 (0.144-0.162)	0.148 (0.139-0.156)	**0.145** (0.136-0.154)	0.146 (0.137-0.155)	0.147 (0.137-0.156)
Confusion matrix	TP: 235, FP: 98, TN: 1282, FN: 339	TP: 209, FP: 66, TN: 1314, FN: 365	TP: 252, FP: 93, TN: 1287, FN: 322	TP: 257, FP: 104, TN: 1276, FN: 317	TP: 261, FP: 116, TN: 1264, FN: 313

Note: Values in parentheses represent 95% confidence intervals estimated using bootstrap resampling (2,000 repetitions).

After applying thresholds optimised using Youden’s index, sensitivity exceeded 70% at the 3-, 5-, and 10-year horizons, while at 1 year it reached 66.4%, as detailed in Table 6.

**Table 6:** Performance of ML models for predicting breast cancer-specific mortality at 1, 3, 5, and 10-year horizons using optimal decision thresholds defined by Youden’s index.

Metrics	Models				
	Logistic	Random Forest	CatBoost	XGBoost	LightGBM
**1-year**				
Accuracy	84.54 (82.65-85.98)	85.06 (83.37-86.59)	86.28 (84.75-87.77)	86.28 (84.80-87.82)	86.49 (84.95-88.02)
Precision	41.16 (35.83-45.98)	42.23 (37.01-47.33)	45.16 (39.65-50.28)	45.13 (39.77-50.41)	45.72 (40.27-50.93)
Sensitivity	66.38 (59.91-72.35)	65.96 (59.58-72.02)	65.53 (59.19-71.43)	65.11 (58.85-71.19)	65.96 (59.58-72.02)
Specificity	87.03 (85.21-88.34)	87.67 (85.95-89.15)	89.12 (87.61-90.55)	89.18 (87.63-90.58)	89.30 (87.81-90.71)
F1-Score	50.81 (45.50-55.24)	51.50 (46.27-56.44)	53.47 (48.25-58.28)	53.31 (48.21-58.01)	54.01 (48.62-58.68)
MCC	43.94 (38.08-48.64)	44.66 (39.09-49.98)	46.84 (41.16-52.13)	46.63 (41.08-51.96)	47.46 (41.61-52.60)
Confusion matrix	TP: 156, FP: 223, TN: 1496, FN: 79	TP: 155, FP: 212, TN: 1507, FN: 80	TP: 154, FP: 187, TN: 1532, FN: 81	TP: 153, FP: 186, TN: 1533, FN: 82	TP: 155, FP: 184, TN: 1535, FN: 80
**3-year**					
Accuracy	68.42 (66.22-70.52)	66.89 (64.84-68.99)	69.96 (67.91-72.06)	67.86 (65.76-69.96)	69.60 (67.50-71.70)
Precision	37.12 (33.85-40.23)	36.29 (33.08-39.33)	38.64 (35.29-41.88)	37.15 (33.91-40.33)	38.54 (35.22-41.80)
Sensitivity	74.88 (70.63-78.95)	78.57 (74.70-82.38)	75.86 (71.61-79.95)	79.06 (75.12-82.97)	77.83 (73.82-81.68)
Specificity	66.73 (64.36-69.14)	63.82 (61.34-66.22)	68.41 (66.10-70.80)	64.92 (62.48-67.32)	67.44 (65.03-69.83)
F1-Score	49.63 (46.14-52.95)	49.65 (46.24-52.95)	51.21 (47.78-54.53)	50.55 (47.07-53.77)	51.55 (48.05-54.87)
MCC	34.21 (29.93-38.33)	34.58 (30.64-38.43)	36.55 (33.47-40.54)	35.93 (31.92-39.82)	37.22 (33.13-41.19)
Confusion matrix	TP: 304, FP: 515, TN: 1033, FN: 102	TP: 319, FP: 560, TN: 988, FN: 87	TP: 308, FP: 489, TN: 1059, FN: 98	TP: 321, FP: 543, TN: 1005, FN: 85	TP: 316, FP: 504, TN: 1044, FN: 90
**5-year**					
Accuracy	72.57 (70.68-74.56)	72.52 (70.73-74.51)	73.44 (71.60-75.44)	74.46 (72.62-76.41)	73.80 (71.95-75.79)
Precision	47.46 (44.13-51.05)	47.51 (44.26-51.03)	48.52 (45.18-52.20)	49.86 (46.28-53.68)	48.98 (45.50-52.63)
Sensitivity	73.44 (69.58-77.38)	76.86 (73.26-80.68)	72.43 (68.50-76.46)	72.03 (68.08-76.20)	72.23 (68.43-76.30)
Specificity	72.27 (69.96-74.56)	71.04 (68.81-73.37)	73.78 (71.55-76.04)	75.29 (73.04-77.48)	74.33 (72.10-76.53)
F1-Score	57.66 (54.50-60.90)	58.72 (55.75-61.94)	58.11 (55.10-61.33)	58.93 (55.87-62.26)	58.37 (55.31-61.75)
MCC	40.75 (36.87-44.89)	42.39 (38.81-46.49)	41.47 (37.70-45.70)	42.75 (38.92-46.91)	41.88 (38.03-46.12)
Confusion matrix	TP: 365, FP: 404, TN: 1053, FN: 132	TP: 382, FP: 422, TN: 1035, FN: 115	TP: 360, FP: 382, TN: 1075, FN: 137	TP: 358, FP: 360, TN: 1097, FN: 139	TP: 359, FP: 374, TN: 1083, FN: 138
**10-year**					
Accuracy	74.51 (72.62-76.36)	74.77 (72.82-76.71)	76.05 (74.16-77.94)	75.69 (73.75-77.58)	76.15 (74.26-78.05)
Precision	54.99 (51.37-58.50)	55.12 (51.53-58.63)	56.97 (53.35-60.45)	56.63 (53.12-60.14)	57.46 (53.75-61.05)
Sensitivity	73.00 (69.31-76.52)	75.96 (72.19-79.53)	75.44 (71.65-79.15)	73.69 (69.91-77.38)	72.47 (68.65-76.32)
Specificity	75.14 (72.93-77.32)	74.28 (71.93-76.53)	76.30 (74.11-78.50)	76.52 (74.32-78.63)	77.68 (75.57-79.83)
F1-Score	62.72 (59.64-65.65)	63.88 (60.72-66.81)	64.92 (61.79-67.85)	64.04 (60.92-67.05)	64.10 (60.94-67.23)
MCC	44.96 (40.79-48.93)	46.61 (42.63-50.61)	48.34 (44.21-52.45)	47.07 (42.94-51.11)	47.30 (43.16-51.48)
Confusion matrix	TP: 419, FP: 343, TN: 1037, FN: 155	TP: 436, FP: 355, TN: 1025, FN: 138	TP: 433, FP: 327, TN: 1053, FN: 141	TP: 423, FP: 324, TN: 1056, FN: 151	TP: 416, FP: 308, TN: 1072, FN: 158

**Note:** Values in parentheses represent 95% confidence intervals estimated using bootstrap resampling. The optimal decision threshold was defined using Youden’s index (J = 0.111) based on the superior-performing 1-year model (LightGBM), which demostrated the lowest Brier Score, and subsequently applied to all models to ensure comparability. For the 3-year prediction, Youden’s index was J = 0.176. For the 5-year and 10-year predictions, Youden’s index values were J = 0.261 and J = 0.340, respectively.

### Comparative analysis and internal model validation

Sensitivity and specificity comparisons based on Youden’s index are reported in the Supplementary Material (S1 and S2 Tables in S1 File). In most time horizons, ensemble-based models showed higher specificity than logistic regression (McNemar’s test, exact p-values reported in S1 and S2 Tables in S1 File), with no significant differences in sensitivity for all-cause and breast cancer-specific mortality.

**Fig 3:**
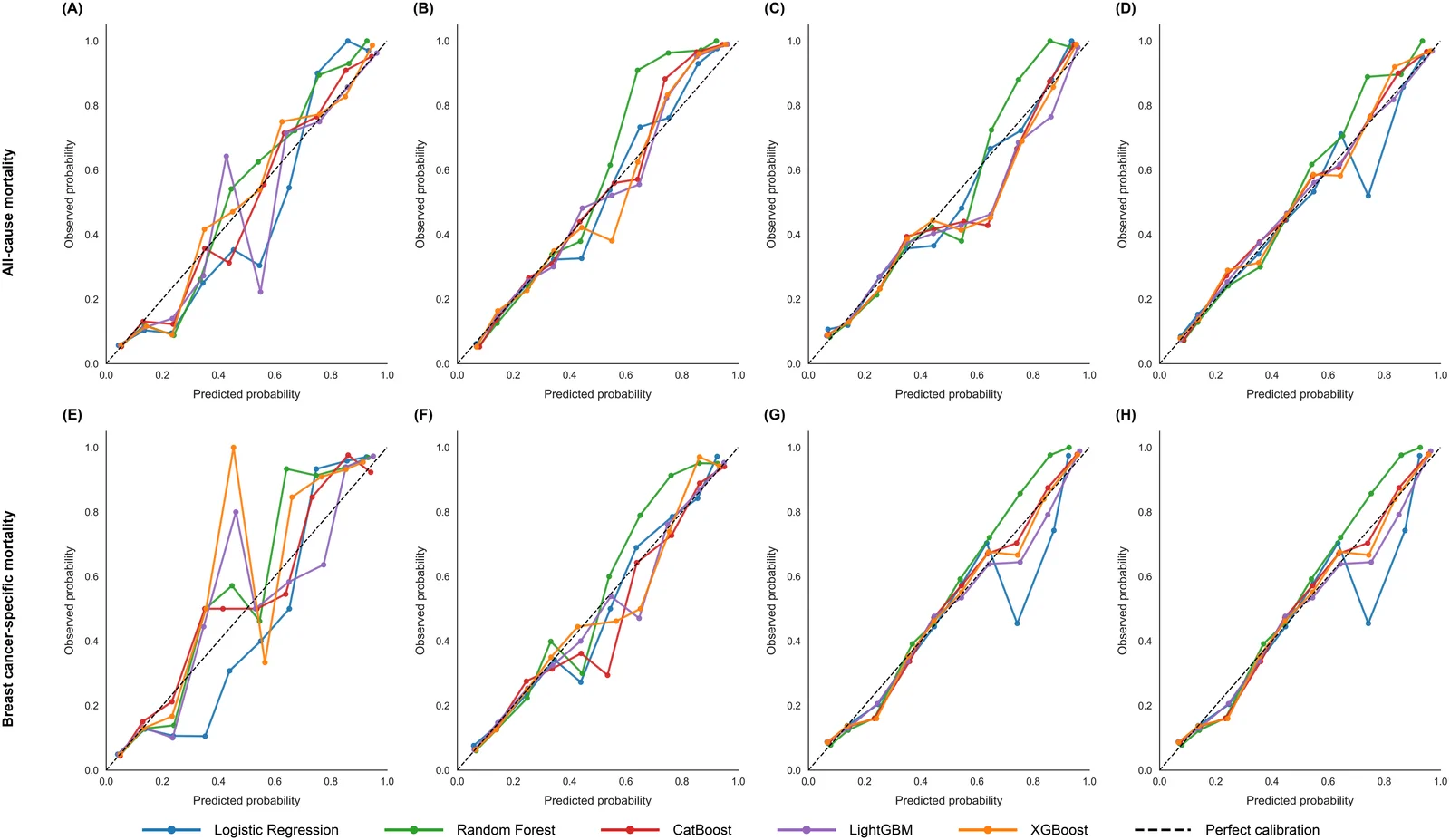
Calibration plots for mortality prediction. Panels A-D correspond to all-cause mortality at 1, 3, 5, and 10 years. Panels E-H correspond to breast cancer-specific mortality at 1, 3, 5, and 10 years.

Internal validity was evaluated using calibration plots and the Brier score with bootstrap resampling (Fig 3; Tables 3 and 5). All models showed good calibration (Brier score < 0.20), with consistently better performance observed for ensemble-based models across all prediction horizons.

Comparative results are presented in the Supplementary Material (S3 Table in S1 File). For all-cause 1-year mortality, CatBoost showed improved calibration relative to logistic regression (ΔBrier score = 0.0027; 95% CI: 0.0004–0.0049; p = 0.0218). At the 3-year horizon, LightGBM demonstrated superior calibration compared with logistic regression (ΔBrier score = 0.0064; 95% CI: 0.0031–0.0096; p < 0.001). For breast cancer-specific mortality at 10 years, CatBoost again showed better calibration than logistic regression (ΔBrier score = 0.0084; 95% CI: 0.0051–0.0118; p < 0.001).

### Importance of predictors

**Fig 4:**
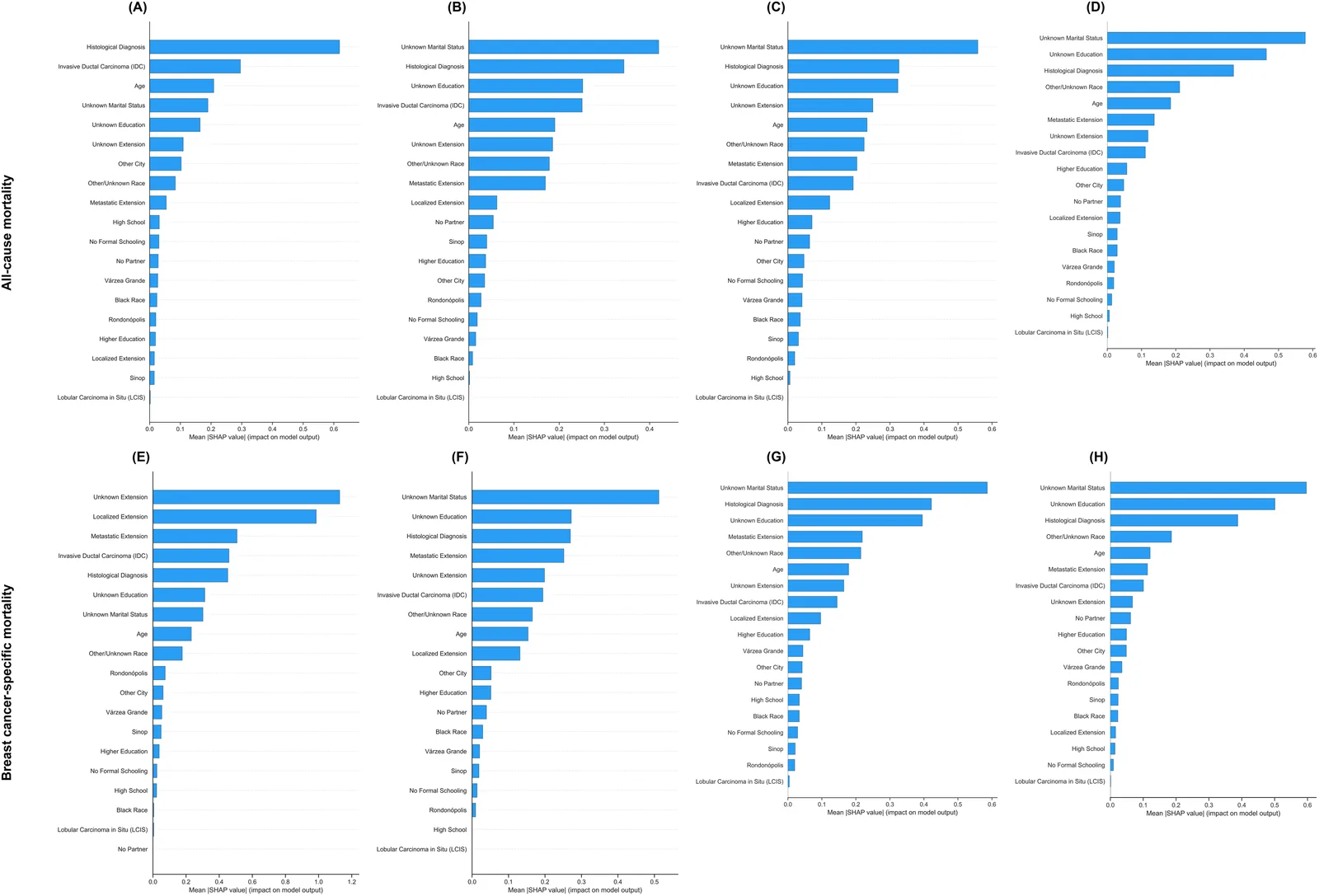
SHAP-based feature importance for the best-performing models across prediction time horizons. Panels A-D show the SHAP feature importance for the models predicting all-cause mortality at 1, 3, 5, and 10 years, respectively. Panels E-H present the corresponding SHAP feature importance rankings for breast cancer-specific mortality at the same prediction horizons.

**Fig 5:**
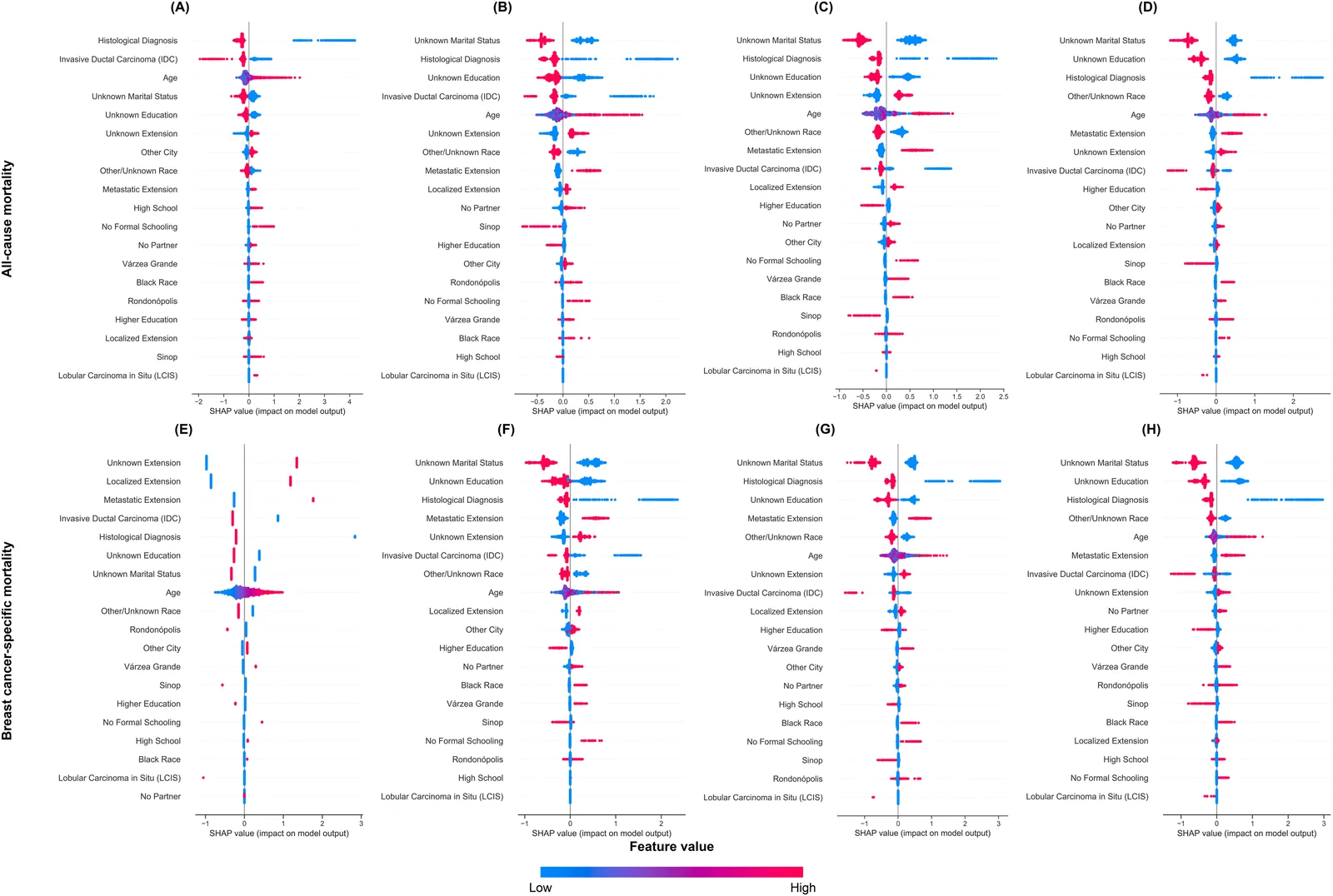
SHAP summary plot illustrating the contribution of each predictor to the mortality models across prediction time horizons. Panels A-D show SHAP feature importance for models predicting all-cause mortality at 1, 3, 5, and 10 years, respectively, while panels E-H present the corresponding feature importance for breast cancer-specific mortality at the same time horizons. Each point represents an individual observation, with its horizontal position indicating the direction and magnitude of the feature’s influence on the model output.

The models with the best AUROC performance were subsequently analysed using the SHAP framework. The most important predictors are presented in Figs 4 and 5. Fig 4 depicts the global importance of the variables, expressed as the mean absolute SHAP value, which reflects the average contribution of each predictor to the model output. Overall, the importance patterns were consistent across all evaluated prediction horizons.

For all-cause mortality, the most influential predictors included histological diagnosis, invasive ductal carcinoma (IDC), age, unknown marital status, unknown stage at diagnosis, race/ethnicity classified as other or unknown, and metastatic stage. For breast cancer–specific mortality, the most relevant predictors included unknown stage at diagnosis, localised stage, metastatic stage, IDC, histological diagnosis, unknown educational level, unknown marital status, and age.

Fig 5 presents the SHAP summary scatter plot, which illustrates both the magnitude and the direction of the impact of each predictor on the estimated risk of mortality. In this plot, each point represents an individual observation, with colour indicating the value of the predictor. Higher SHAP values correspond to a greater influence of the predictor on the model’s predicted outcome.

### Decision curve analysis of model clinical utility

**Fig 6:**
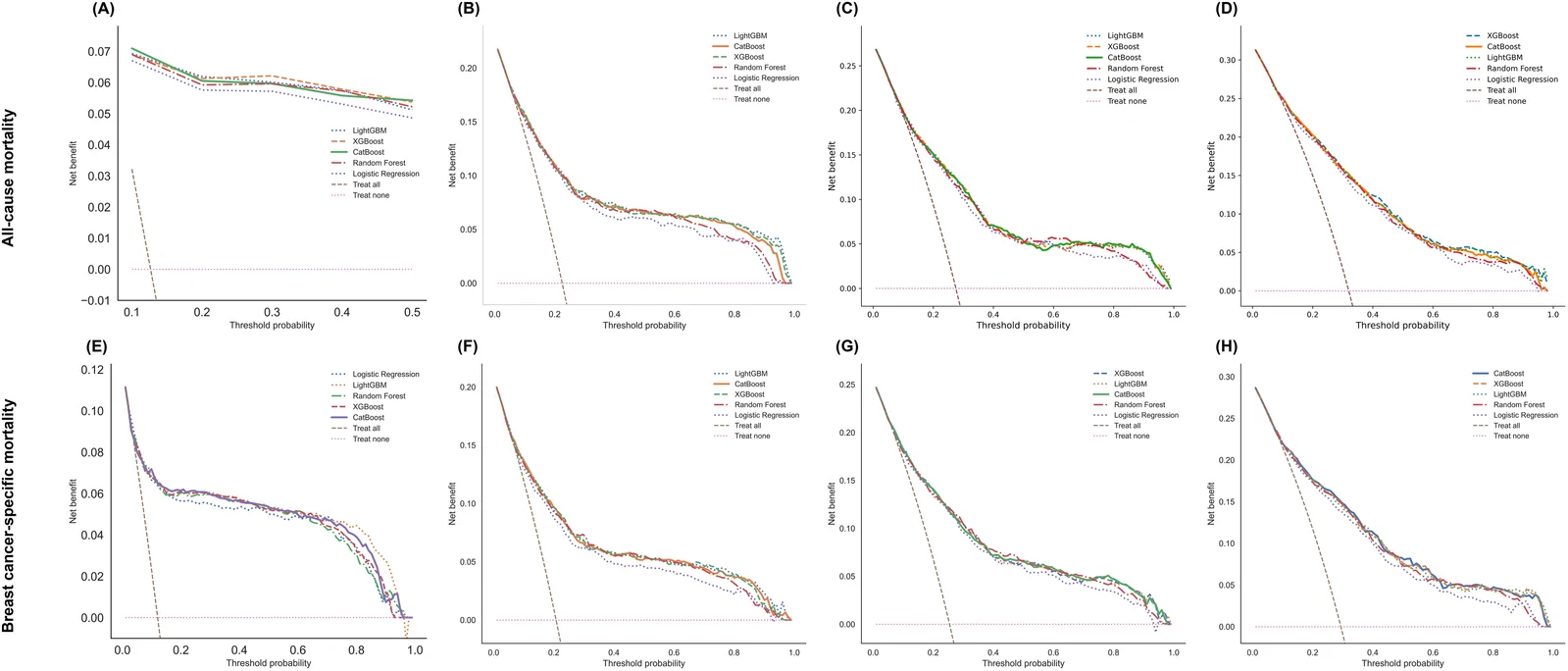
Decision curve analysis (DCA) plots illustrating the clinical utility of the evaluated models across prediction time horizons. Panels A-D show the net benefit for models predicting all-cause mortality at 1, 3, 5, and 10 years, respectively, while panels E-H present the corresponding net benefit for breast cancer-specific mortality at the same time horizons.

DCA showed that all models provided a positive net benefit across a broad range of threshold probabilities compared with the “treat all” and “treat none” strategies. Gradient boosting models showed slightly higher net benefit, particularly at intermediate threshold probabilities (approximately 0.2-0.6) (Fig 6).

## Discussion

In this population-based cohort of women diagnosed with breast cancer in Mato Grosso, Brazil, ML models showed consistent performance for predicting both all-cause and breast cancer-specific mortality across 1-, 3-, 5-, and 10-year horizons. Overall, gradient-boosting models (LightGBM, XGBoost, and CatBoost) achieved a stable balance between discrimination and calibration, which reinforces the suitability of ensemble tree-based methods for prognostic modelling using routinely collected cancer registry data.

Our findings are consistent with previous studies showing that ML approaches can match or outperform conventional regression in breast cancer survival prediction, particularly when the relationships between predictors and outcomes are non-linear and include complex interactions [17,18]. However, much of the existing literature originates from high-income settings, where clinical data tend to be more complete and care pathways more standardised [34]. By contrast, Mato Grosso reflects a context of geographic barriers, heterogeneous access to oncology services, and substantial missingness in registry variables, features common to many regions of Brazil and other LMICs [2,6,35]. Demonstrating robust performance under these real-world constraints supports the feasibility of ML-based risk stratification using administrative and registry data [36]. Discrimination declined modestly as the prediction horizon increased, a pattern widely reported in long-term prognostic modelling and plausibly related to competing risks, treatment heterogeneity, and unmeasured time-varying determinants [34,37]. Nonetheless, AUROC values remained in a clinically informative range across follow-up. Beyond discrimination, decision curve analysis suggests that the models may provide clinical value across a wide range of risk thresholds, indicating potential to support more refined decision-making than default strategies. The slightly greater utility observed for gradient boosting approaches further points to their advantage in identifying patients within clinically actionable risk ranges.

Gradient boosting is particularly effective for structured datasets in which predictor effects are heterogeneous and non-linear. Methods such as XGBoost and LightGBM implement regularisation and handle missing values pragmatically, often improving robustness when stage or socioeconomic indicators are incompletely captured, an important feature for population registries in Brazil [21,23]. At the same time, performance similarities among several models highlight that there is no universally optimal algorithm, consistent with the “no free lunch” principle, and that model selection should remain empirical and context-specific, particularly when the goal is deployment rather than retrospective benchmarking [38].

Mortality prediction in breast cancer is inherently imbalanced, especially at shorter horizons, and reliance on a single metric such as AUROC can obscure clinically relevant shortcomings. Recent methodological work highlights the complementary roles and limitations of AUROC, AUPRC, and threshold-dependent metrics, cautioning against simplistic interpretations of performance under class imbalance [39–41]. In this study, the use of multiple performance measures beyond AUROC, including overall performance metrics such as the Brier score and calibration plots assessments, strengthens interpretability and aligns with best-practice recommendations for clinical prediction modelling [37,40].

Explainable ML approaches based on SHAP provide a unified framework for decomposing model predictions into feature-level contributions and have become increasingly adopted in oncology research [30]. In the present study, predictor importance patterns were broadly consistent across endpoints and prediction horizons, reinforcing the robustness of the identified signals.

Key predictors included age at diagnosis, cancer stage (localised, metastatic, and unknown), histological diagnosis, IDC, unknown marital status, skin colour (race) classified as other or unknown, and unknown educational level. These findings are largely concordant with previous studies applying ML-based classification and survival models in breast cancer, which have similarly highlighted the central role of tumour stage, age, and histopathological characteristics, while also identifying sociodemographic and access-related variables as relevant contributors to prediction performance [15,42,43].

The consistent influence of educational level and marital status underscores the importance of social determinants of health and care access pathways in shaping breast cancer outcomes, even within the context of a universal health system. Such variables may act as proxies for differences in health literacy, care-seeking behaviour, diagnostic timeliness, and continuity of treatment, rather than reflecting tumour biology alone [43,44]. These observations align with epidemiological evidence demonstrating persistent social gradients in cancer survival across diverse settings [45–47]. Notably, categories recorded as “unknown” also emerged as relevant predictors, suggesting that unrecorded information in administrative databases may reflect disparities in data quality, access to care, or clinical management processes. Rather than representing random missingness, these categories may indicate systematic differences in documentation practices, healthcare utilization, or delays in diagnosis and treatment. Thus, their contribution reinforces the importance of considering data completeness as an informative dimension, particularly in population-based registries, where such absence may reflect structural factors influencing patient outcomes.

Importantly, SHAP-based analyses revealed stable and clinically plausible patterns across different models, thereby increasing confidence in their transparency and reliability [48].This level of interpretability is essential for clinical acceptance and directly addresses one of the most commonly cited barriers to the implementation of ML tools in oncology practice, namely the lack of trust and explainability in model-driven decision support systems.

From a clinical perspective, these models may support early identification of women at high risk of mortality, enabling intensified follow-up, timely referral to specialised oncology services, and prioritisation of diagnostic and therapeutic interventions [49]. However, these models are intended to complement, rather than replace, established clinical staging systems and physician judgment.

The use of a unified decision threshold based on Youden’s index provides a pragmatic balance between sensitivity and specificity and facilitates comparability across models and prediction horizons. At the health system level, ML-based risk stratification may inform resource allocation within the SUS, particularly in settings with limited oncology infrastructure [50], offering a scalable and cost-effective approach based on routinely collected data. Despite these potential applications, challenges remain for clinical implementation, including limited real-time applicability, lack of automated data integration, and difficulties in translating population-based predictions into individual clinical decision-making.

This study leverages linked cancer registry and mortality data to enable long-term follow-up and the evaluation of both all-cause and breast cancer-specific mortality across multiple time horizons. A key strength is the comprehensive evaluation framework, integrating discrimination, calibration, threshold-dependent metrics, and formal model comparisons, which highlights the added value of gradient boosting methods over logistic regression under routine-data constraints. The use of explainable ML further enhances interpretability and supports clinically plausible and equity-oriented model assessment.

However, this study has limitations inherent to registry-based research. The absence or incompleteness of key clinical variables in the RCBP, such as stage, detailed tumour characteristics, treatment information, comorbidities, molecular subtype, and disease recurrence, may have affected predictive performance. In addition, some sociodemographic and clinical variables, including marital status, education level, and stage, contained unrecorded or poorly captured information in administrative data, with categories coded as “unknown,” which likely reflect gaps in data recording and disparities in access to diagnostic services rather than true patient characteristics.

Variability in data collection across centres in Mato Grosso may have affected data quality, and deterministic linkage between the RCBP and the SIM may be subject to missed or false matches, potentially leading to outcome misclassification. Mortality was also defined as a binary outcome at prespecified prediction horizons (1, 3, 5, and 10 years). Although this approach facilitates clinically interpretable risk prediction, it simplifies the underlying time-to-event process and does not fully account for censoring, which may have influenced predictive performance, particularly at longer prediction horizons. The exclusion of male patients, due to their low frequency and distinct clinical and biological behaviour, limits applicability to women. The absence of external validation represents an important limitation of this study. Although the models demonstrated robust internal performance through independent test set evaluation, stratified five-fold cross-validation, bootstrap resampling, discrimination, calibration, and overall prediction error analyses, their performance in independent populations remains unknown. Consequently, the findings should be interpreted primarily in the context of women diagnosed with breast cancer in Mato Grosso. Future multicentre studies involving independent population-based cancer registries are needed to evaluate the transportability and generalisability of these models before broader implementation in other settings. Emerging privacy-preserving approaches, such as federated learning, may facilitate future multicentre validation while addressing ethical and regulatory constraints on sharing individual-level health data. Future research should prioritise multicentre external validation, prospective evaluation, and the development of automated data integration pipelines. It should also explore time-updated models and the integration of longitudinal clinical, imaging-derived, and genomic data to improve predictive performance and clinical utility.

## Conclusion

ML models showed good and stable performance in predicting all-cause and breast cancer-specific mortality among women in Mato Grosso, Brazil, using routine population-based data. Gradient boosting algorithms, particularly CatBoost, XGBoost, and LightGBM, demonstrated superior discrimination and calibration across prediction horizons. Age at diagnosis, cancer stage, histopathological type, and marital status emerged as key predictors, highlighting the combined influence of clinical and social factors on survival. These findings suggest that ML-based models may support early identification of high-risk patients and inform risk stratification and follow-up planning within the Brazilian SUS, particularly in resource-constrained settings. Nevertheless, because the models have not yet been externally validated, their applicability to populations beyond Mato Grosso remains uncertain. External validation in independent populations and prospective evaluation are therefore warranted before broader clinical or public health implementation.

## Supporting information

S1 File S1 Figs and S4 Tables. (DOCX)

## References

[1] Bray F, Laversanne M, Sung H, Ferlay J, Siegel RL, Soerjomataram I. Global cancer statistics 2022: GLOBOCAN estimates of incidence and mortality worldwide for 36 cancers in 185 countries. CA Cancer J Clin. 2024;74:229–63. 10.3322/caac.21834 38572751

[2] Arnold M, Morgan E, Rumgay H, Mafra A, Singh D, Laversanne M, et al. Current and future burden of breast cancer: global statistics for 2020 and 2040. Breast. 2022;66:15–23. doi: 10.1016/j.breast.2022.08.010 36084384 PMC9465273

[3] Guo L, Kong D, Liu J, Zhan L, Luo L, Zheng W, et al. Breast cancer heterogeneity and its implication in personalized precision therapy. Exp Hematol Oncol. 2023;12(1):3. doi: 10.1186/s40164-022-00363-1 36624542 PMC9830930

[4] Cecilio AP, Takakura ET, Jumes JJ, Dos Santos JW, Herrera AC, Victorino VJ, et al. Breast cancer in Brazil: epidemiology and treatment challenges. Breast Cancer (Dove Med Press). 2015;7:43–9. doi: 10.2147/BCTT.S50361 25678813 PMC4317062

[5] Silva JDDE, de Oliveira RR, da Silva MT, Carvalho MD de B, Pedroso RB, Pelloso SM. Breast cancer mortality in young women in Brazil. Front Oncol. 2021;10:569933. doi: 10.3389/fonc.2020.569933 33585192 PMC7874105

[6] Gonzaga CMR, Freitas-Junior R, Souza MR, Curado MP, Freitas NMA. Disparities in female breast cancer mortality rates between urban centers and rural areas of Brazil: ecological time-series study. Breast. 2014;23(2):180–7. doi: 10.1016/j.breast.2014.01.006 24503143

[7] Dos Santos Figueiredo FW, Adami F. Effects of the high-inequality of income on the breast cancer mortality in Brazil. Sci Rep. 2019;9(1):4173. doi: 10.1038/s41598-019-41012-8 30862862 PMC6414632

[8] Xavier SP, Galvão ND, das Neves MAB, da Silva KM, Almeida A de QN, Silva AMC da. Nomogram model for predicting the long-term prognosis of cervical cancer patients: a population-based study in Mato Grosso, Brazil. BMC Cancer. 2025;25(1):684. doi: 10.1186/s12885-025-14056-5 40229703 PMC11995657

[9] das Neves MAB, Galvão ND, de Lima FC da S, Oliveria JFP, Xavier SP, da Silva KM. Trend in cancer incidence in Mato Grosso and its health regions, Brazil, 2001-2018. Arch Public Health. 2025;83:87. doi: 10.1186/s13690-025-01503-9 PMC11960033 40170090

[10] Oliveira JC de S, Castelo LM, Soares MR, Magalhães AS, Eustáquio DM da C, Navarro-Silva JP, et al. Incidence and mortality by the main types of cancer in the city of Cuiabá, Mato Grosso, between the years of 2008 and 2016. Rev Bras Epidemiol. 2022;25(Supl 1):e220011. doi: 10.1590/1980-549720220011.supl.1 35766768

[11] Wishart GC, Bajdik CD, Azzato EM, Dicks E, Greenberg DC, Rashbass J, et al. A population-based validation of the prognostic model PREDICT for early breast cancer. Eur J Surg Oncol. 2011;37(5):411–7. doi: 10.1016/j.ejso.2011.02.001 21371853

[12] Dankwa-Mullan I, Weeraratne D. Artificial intelligence and machine learning technologies in cancer care: addressing disparities, bias, and data diversity. Cancer Discov. 2022;12(6):1423–7. doi: 10.1158/2159-8290.CD-22-0373 35652218 PMC9662931

[13] das Neves MAB, Galvão ND, de Lima FC da S, Oliveria JFP, Xavier SP, da Silva AMC. Factors associated with advanced diagnosis of cervical cancer: a hospital-based retrospective study in a state of the Brazilian Legal Amazon. 2025. 10.3332/ecancer.2025.2045 PMC12950892 41778184

[14] Hasnis E, Avizohar O, Half EE, Aran D. Machine learning for cancer risk stratification: a bi-directional approach to screening. American Society of Clinical Oncology; 2025.

[15] Hamedi SZ, Emami H, Khayamzadeh M, Rabiei R, Aria M, Akrami M, et al. Application of machine learning in breast cancer survival prediction using a multimethod approach. Sci Rep. 2024;14(1):30147. doi: 10.1038/s41598-024-81734-y 39627494 PMC11615207

[16] Botlagunta M, Botlagunta MD, Myneni MB, Lakshmi D, Nayyar A, Gullapalli JS, et al. Classification and diagnostic prediction of breast cancer metastasis on clinical data using machine learning algorithms. Sci Rep. 2023;13(1):485. doi: 10.1038/s41598-023-27548-w 36627367 PMC9831019

[17] Ganggayah MD, Taib NA, Har YC, Lio P, Dhillon SK. Predicting factors for survival of breast cancer patients using machine learning techniques. BMC Med Inform Decis Mak. 2019;19(1):48. doi: 10.1186/s12911-019-0801-4 30902088 PMC6431077

[18] Silva G, Duarte L, Shirassu M, Peres S, de Moraes M, Chiavegatto Filho A. Machine learning for longitudinal mortality risk prediction in patients with malignant neoplasm in São Paulo, Brazil. Artificial Intelligence in the Life Sciences. 2023;3:100061. doi: 10.1016/j.ailsci.2023.100061

[19] Lima FC da S de, Souza B da SN de, Oliveira JFP, Galvão ND, Souza PCF de. Cervical cancer specific survival in Grande Cuiabá, Mato Grosso State, Brazil. Rev Bras Epidemiol. 2022;25(Supl 1):e220017. doi: 10.1590/1980-549720220017.supl.1 35766774

[20] Fritz A, Percy C, Jack A, Shanmugaratnam K, Sobin L, Parkin DM, et al. International classification of diseases for oncology. World Health Organization; 2000.

[21] Chen T, Guestrin C. XGBoost: a scalable tree boosting system. In: Proceedings of the 22nd ACM SIGKDD International conference on knowledge discovery and data mining. 2016. 785–94.

[22] Prokhorenkova L, Gusev G, Vorobev A, Dorogush AV, Gulin A. CatBoost: unbiased boosting with categorical features. Adv Neural Inf Process Syst. 2018;31.

[23] Ke G, Meng Q, Finley T, Wang T, Chen W, Ma W. Lightgbm: a highly efficient gradient boosting decision tree. Adv Neural Inf Process Syst. 2017;30.

[24] Roy P, Hasan M, Islam MR, Uddin MP. Interpretable artificial intelligence (AI) for cervical cancer risk analysis leveraging stacking ensemble and expert knowledge. Digit Health. 2025;11. doi: 10.1177/20552076251327945 40144051 PMC11938887

[25] Murali N, Kucukkaya A, Petukhova A, Onofrey J, Chapiro J. Supervised machine learning in oncology: a clinician’s guide. Dig Dis Interv. 2020;4(1):73–81. doi: 10.1055/s-0040-1705097 32869010 PMC7456427

[26] Bradshaw TJ, Huemann Z, Hu J, Rahmim A. A guide to cross-validation for artificial intelligence in medical imaging. Radiol Artif Intell. 2023;5(e220232). 10.1148/ryai.220232 PMC10388213 37529208

[27] Rainio O, Teuho J, Klén R. Evaluation metrics and statistical tests for machine learning. Sci Rep. 2024;14(1):6086. doi: 10.1038/s41598-024-56706-x 38480847 PMC10937649

[28] Yang W, Jiang J, Schnellinger EM, Kimmel SE, Guo W. Modified Brier score for evaluating prediction accuracy for binary outcomes. Stat Methods Med Res. 2022;31(12):2287–96. doi: 10.1177/09622802221122391 36031854 PMC9691523

[29] Steyerberg EW, Vickers AJ, Cook NR, Gerds T, Gonen M, Obuchowski N, et al. Assessing the performance of prediction models: a framework for traditional and novel measures. Epidemiology. 2010;21(1):128–38. doi: 10.1097/EDE.0b013e3181c30fb2 20010215 PMC3575184

[30] Lundberg SM, Lee SI. A unified approach to interpreting model predictions. Adv Neural Inf Process Syst. 2017;30.

[31] Ponce-Bobadilla AV, Schmitt V, Maier CS, Mensing S, Stodtmann S. Practical guide to SHAP analysis: Explaining supervised machine learning model predictions in drug development. Clin Transl Sci. 2024;17(11):e70056. doi: 10.1111/cts.70056 39463176 PMC11513550

[32] Zhao L, Leng Y, Hu Y, Xiao J, Li Q, Liu C, et al. Understanding decision curve analysis in clinical prediction model research. Postgraduate Medical Journal. 2024;100(1185):512–5. doi: 10.1093/postmj/qgae027 38453146

[33] Shreffler J, Huecker MR. Survival analysis. 2020.

[34] Parikh RB, Manz C, Chivers C, Regli SH, Braun J, Draugelis ME, et al. Machine learning approaches to predict 6-month mortality among patients with cancer. JAMA Network Open. 2019;2(e1915997). doi: 10.1001/jamanetworkopen.2019.15997 PMC6822091 31651973

[35] Bizuayehu HM, Ahmed KY, Kibret GD, Dadi AF, Belachew SA, Bagade T. Global disparities of cancer and its projected burden in 2050. JAMA Netw Open. 2024;7:e2443198. doi: 10.1001/jamanetworkopen.2024.43198 PMC11539015 39499513

[36] Gupta S, Tran T, Luo W, Phung D, Kennedy RL, Broad A, et al. Machine-learning prediction of cancer survival: a retrospective study using electronic administrative records and a cancer registry. BMJ Open. 2014;4(3):e004007. doi: 10.1136/bmjopen-2013-004007 24643167 PMC3963101

[37] Steyerberg EW, Vickers AJ. Decision curve analysis: a discussion. Med Decis Making. 2008;28(1):146–9. doi: 10.1177/0272989X07312725 18263565 PMC2577563

[38] Wolpert DH, Macready WG. No free lunch theorems for optimization. IEEE Trans Evol Computat. 1997;1(1):67–82. doi: 10.1109/4235.585893

[39] Hicks SA, Strümke I, Thambawita V, Hammou M, Riegler MA, Halvorsen P, et al. On evaluation metrics for medical applications of artificial intelligence. Sci Rep. 2022;12(1):5979. doi: 10.1038/s41598-022-09954-8 35395867 PMC8993826

[40] Van Calster B, Collins GS, Vickers AJ, Wynants L, Kerr KF, Barreñada L, et al. Evaluation of performance measures in predictive artificial intelligence models to support medical decisions: overview and guidance. Lancet Digit Health. 2025;7(12):100916. doi: 10.1016/j.landig.2025.100916 41391983

[41] Saito T, Rehmsmeier M. The precision-recall plot is more informative than the ROC plot when evaluating binary classifiers on imbalanced datasets. PLoS One. 2015;10(3):e0118432. doi: 10.1371/journal.pone.0118432 25738806 PMC4349800

[42] Baidoo TG, Rodrigo H. Data-driven survival modeling for breast cancer prognostics: a comparative study with machine learning and traditional survival modeling methods. PLoS One. 2025;20(4):e0318167. doi: 10.1371/journal.pone.0318167 40262081 PMC12014147

[43] Park SW, Park Y-L, Lee E-G, Chae H, Park P, Choi D-W, et al. Mortality prediction modeling for patients with breast cancer based on explainable machine learning. Cancers (Basel). 2024;16(22):3799. doi: 10.3390/cancers16223799 39594754 PMC11592669

[44] Moncada-Torres A, van Maaren MC, Hendriks MP, Siesling S, Geleijnse G. Explainable machine learning can outperform Cox regression predictions and provide insights in breast cancer survival. Sci Rep. 2021;11(1):6968. doi: 10.1038/s41598-021-86327-7 33772109 PMC7998037

[45] Sun W, Jiang Y-Z, Liu Y-R, Ma D, Shao Z-M. Nomograms to estimate long-term overall survival and breast cancer-specific survival of patients with luminal breast cancer. Oncotarget. 2016;7(15):20496–506. doi: 10.18632/oncotarget.7975 26967253 PMC4991470

[46] Teng L, Zhang Z, Du J, Dong Y, Tao W. Nomogram construction and survival analysis in T3N0M0 breast cancer: a SEER population-based analysis. Sci Rep. 2025;15(1):25194. doi: 10.1038/s41598-025-08518-w 40651990 PMC12255696

[47] Yu Z-H, Lin Y, Wu P-S, Lee C-H, Chou C-P. A prognostic nomogram for predicting breast cancer survival based on mammography and AJCC staging. Heliyon. 2024;10(5):e27072. doi: 10.1016/j.heliyon.2024.e27072 38449621 PMC10915383

[48] Ghasemi A, Hashtarkhani S, Schwartz DL, Shaban-Nejad A. Explainable artificial intelligence in breast cancer detection and risk prediction: a systematic scoping review. Cancer Innov. 2024;3(5):e136. doi: 10.1002/cai2.136 39430216 PMC11488119

[49] Zhang B, Shi H, Wang H. Machine learning and AI in cancer prognosis, prediction, and treatment selection: a critical approach. J Multidiscip Healthc. 2023;16:1779–91. doi: 10.2147/JMDH.S410301 PMC10312208 37398894

[50] Susanto AP, Lyell D, Widyantoro B, Berkovsky S, Magrabi F. Effects of machine learning-based clinical decision support systems on decision-making, care delivery, and patient outcomes: a scoping review. J Am Med Inform Assoc. 2023;30(12):2050–63. doi: 10.1093/jamia/ocad180 37647865 PMC10654852

