# Supplementary material for: Machine learning for predicting mortality in women diagnosed with breast cancer in the State of Mato Grosso, Brazil using linked population-based cancer registry and mortality data
Source: PLoS One. 2026 Sep 18;21(9):e0356621. doi: 10.1371/journal.pone.0356621 (PMC13588536; doi:10.1371/journal.pone.0356621)
Supplement: Supplementary file 1 [file pone.0356621.s001.docx]

**Supplementary material**

**S.Fig. 1.** Flowchart of case selection from the Mato Grosso Population-Based Cancer Registry (RCBP, 2001-2018) and the Mortality Information System (SIM, 2001-2022). After deduplication and linkage, 91,112 incident cancer cases were identified. Of these, 7,953 were breast cancer cases (ICD-10: C50.0-C50.9). After excluding records with non-applicable staging (n = 1), missing age (n = 18), missing diagnostic method (n = 51), and male sex (n = 68), the final sample included 7,815 breast cancer cases.

**S. Fig. 2.** Schematic overview of the analytical pipeline. Data were preprocessed using encoding and normalization procedures, followed by stratified train-test splitting. Multiple machine learning models were developed and evaluated using using AUROC, accuracy, precision, sensitivity, specificity, F1-score, MCC, and Brier score. Statistical comparisons included DeLong and McNemar tests, with confidence intervals estimated via bootstrap resampling. Model robustness was evaluated using ROC curve perturbation with Gaussian noise.

**Table S-1.** Statistical comparison of ML models using McNemar’s χ² test across accuracy, sensitivity, and specificity for all-cause mortality

| **Comparison** | **χ² (global accuracy)** | **p-value*** | **χ² (sensitivity)** | **p-value** | **χ² (specificity)** | **p-value** |
| --- | --- | --- | --- | --- | --- | --- |
|  | **all-cause mortality** | | | | |  |
|  |  |  | **1 year** |  |  |  |
| LGBM vs XGB | 3.8409 | 0.0500 | 0.0000 | 1.0000 | 5.0256 | 0.0250 |
| LGBM vs RF | 42.4512 | 0.0000 | 3.1250 | 0.0771 | 37.9595 | 0.0000 |
| LGBM vs LR | 43.5220 | 0.0000 | 1.2308 | 0.2673 | 41.7515 | 0.0000 |
| LGBM vs CatB | 3.0118 | 0.0827 | 3.2727 | 0.0704 | 1.0946 | 0.2955 |
| XGB vs RF | 30.6818 | 0.0000 | 4.0000 | 0.0455 | 25.3333 | 0.0000 |
| XGB vs LR | 33.8855 | 0.0000 | 1.5625 | 0.2113 | 31.7400 | 0.0000 |
| XGB vs CatB | 0.0421 | 0.8374 | 3.5000 | 0.0614 | 0.1975 | 0.6567 |
| RF vs LR | 5.1280 | 0.0235 | 0.0000 | 1.0000 | 6.1224 | 0.0133 |
| RF vs CatB | 20.2759 | 0.0000 | 0.0000 | 1.0000 | 24.3289 | 0.0000 |
| LR vs CatB | 25.0435 | 0.0000 | 0.0455 | 0.8312 | 29.6000 | 0.0000 |
|  |  |  | **3 years** |  |  |  |
| LGBM vs XGB | 2.8333 | 0.0923 | 2.8929 | 0.0890 |  |  |
| LGBM vs RF | 326.7315 | 0.0000 | 232.9201 | 0.0000 | 120.0192 | 0.0000 |
| LGBM vs LR | 0.3293 | 0.5661 | 0.1667 | 0.6831 | 2.4231 | 0.1196 |
| LGBM vs CatB | 4.4944 | 0.0340 | 0.0000 | 1.0000 | 5.4697 | 0.0193 |
| XGB vs RF | 310.8957 | 0.0000 | 224.6413 | 0.0000 | 113.5087 | 0.0000 |
| XGB vs LR | 3.2257 | 0.0725 | 0.5682 | 0.4510 | 2.4231 | 0.1196 |
| XGB vs CatB | 11.9339 | 0.0006 | 3.4483 | 0.0633 | 7.9239 | 0.0049 |
| RF vs LR | 340.9138 | 0.0000 | 230.5851 | 0.0000 | 133.4767 | 0.0000 |
| RF vs CatB | 349.8357 | 0.0000 | 233.9100 | 0.0000 | 138.1778 | 0.0000 |
| LR vs CatB | 0.4739 | 0.4912 | 0.3902 | 0.5322 | 0.1471 | 0.7014 |
|  |  |  | **5 years** |  |  |  |
| LGBM vs XGB | 1.7297 | 0.1884 | 0.0000 | 1.0000 | 3.5588 | 0.0592 |
| LGBM vs RF | 27.6702 | <0.001 | 0.0000 | 1.0000 | 25.5000 | <0.001 |
| LGBM vs LR | 4.4768 | 0.0344 | 1.4222 | 0.2330 | 7.6556 | 0.0057 |
| LGBM vs CatB | 0.2807 | 0.5962 | 0.0000 | 1.0000 | 0.2462 | 0.6198 |
| XGB vs RF | 22.9091 | 0.0000 | 0.0455 | 0.8312 | 18.5488 | 0.0000 |
| XGB vs LR | 2.0069 | 0.1566 | 1.6875 | 0.1939 | 3.3379 | 0.0677 |
| XGB vs CatB | 0.2250 | 0.6353 | 0.0625 | 0.8026 | 0.6792 | 0.4098 |
| RF vs LR | 4.3308 | 0.0374 | 2.0250 | 0.1547 | 1.9248 | 0.1653 |
| RF vs CatB | 28.9863 | 0.0000 | 0.0455 | 0.8312 | 27.4805 | 0.0000 |
| LR vs CatB | 3.1056 | 0.0780 | 2.1316 | 0.1443 | 6.3712 | 0.0116 |
|  |  |  | **10 years** |  |  |  |
| LGBM vs XGB | 3.2258 | 0.0725 | 0.1000 | 0.7518 | 6.8571 | 0.0088 |
| LGBM vs RF | 4.0000 | 0.0455 | 1.7143 | 0.1904 | 2.0167 | 0.1556 |
| LGBM vs LR | 30.6225 | 0.0000 | 4.3636 | 0.0367 | 25.6356 | 0.0000 |
| LGBM vs CatB | 8.4912 | 0.0036 | 4.9231 | 0.0265 | 3.8409 | 0.0500 |
| XGB vs RF | 11.0658 | 0.0009 | 0.7619 | 0.3827 | 10.4727 | 0.0012 |
| XGB vs LR | 42.7466 | 0.0000 | 3.0303 | 0.0817 | 40.9204 | 0.0000 |
| XGB vs CatB | 2.2407 | 0.1344 | 7.6923 | 0.0055 | 0.0000 | 1.0000 |
| RF vs LR | 16.9085 | 0.0000 | 0.6944 | 0.4047 | 17.4434 | 0.0000 |
| RF vs CatB | 28.0167 | 0.0000 | 10.2273 | 0.0014 | 16.4474 | 0.0001 |
| LR vs CatB | 51.1173 | 0.0000 | 11.0250 | 0.0009 | 39.0246 | 0.0000 |

**Table S-2.** Statistical comparison of ML models using McNemar’s χ² test across accuracy, sensitivity, and specificity for breast cancer–specific mortality

|  | χ² (global accuracy) | p-value* | χ² (sensitivity) | p-value | χ² (specificity) | p-value |
| --- | --- | --- | --- | --- | --- | --- |
| Comparison | **Breast cancer–specific mortality** | | | | | |
|  |  |  | **1 year** |  |  |  |
| LGBM vs XGB | 0.0139 | 0.9062 | 0.0625 | 0.8026 | 0.0179 | 0.8937 |
| LGBM vs RF | 6.5089 | 0.0107 | 0.9615 | 0.3268 | 5.1279 | 0.0235 |
| LGBM vs LR | 9.2356 | 0.0024 | 5.6250 | 0.0177 | 4.4768 | 0.0344 |
| LGBM vs CatB | 0.0091 | 0.9240 | 0.0500 | 0.8231 | 0.0111 | 0.9161 |
| XGB vs RF | 9.3462 | 0.0022 | 0.6429 | 0.4227 | 8.2656 | 0.0040 |
| XGB vs LR | 9.5351 | 0.0020 | 4.0238 | 0.0449 | 5.4825 | 0.0192 |
| XGB vs CatB | 0.0111 | 0.9161 | 0.0833 | 0.7728 | 0.1154 | 0.7341 |
| RF vs LR | 1.2484 | 0.2639 | 2.0250 | 0.1547 | 0.1368 | 0.7115 |
| RF vs CatB | 6.1275 | 0.0133 | 1.5625 | 0.2113 | 4.1977 | 0.0405 |
| LR vs CatB | 7.5117 | 0.0061 | 5.6250 | 0.0177 | 3.3295 | 0.0680 |
|  |  |  | **3 years** |  |  |  |
| LGBM vs XGB | 21.0114 | 0.0000 | 1.4545 | 0.2278 | 18.7532 | 0.0000 |
| LGBM vs RF | 27.3496 | 0.0000 | 0.3077 | 0.5791 | 27.5000 | 0.0000 |
| LGBM vs LR | 0.0000 | 1.0000 | 3.5588 | 0.0592 | 0.5236 | 0.4693 |
| LGBM vs CatB | 5.9753 | 0.0145 | 3.0625 | 0.0801 | 0.1667 | 0.6831 |
| XGB vs RF | 3.0154 | 0.0825 | 0.1000 | 0.7518 | 4.6545 | 0.0310 |
| XGB vs LR | 8.8402 | 0.0029 | 8.8276 | 0.0030 | 3.8368 | 0.0501 |
| XGB vs CatB | 38.5487 | 0.0000 | 8.4706 | 0.0036 | 25.6889 | 0.0000 |
| RF vs LR | 17.7602 | 0.0000 | 6.7586 | 0.0093 | 11.5928 | 0.0007 |
| RF vs CatB | 45.5625 | 0.0000 | 5.2632 | 0.0218 | 31.7195 | 0.0000 |
| LR vs CatB | 2.1202 | 0.1454 | 0.2812 | 0.5959 | 0.1989 | 0.6556 |
|  |  |  | **5 years** |  |  |  |
| LGBM vs XGB | 4.5581 | 0.0328 | 0.0000 | 1.0000 | 4.9706 | 0.0258 |
| LGBM vs RF | 42.6087 | 0.0000 | 15.6129 | 0.0001 | 26.2976 | 0.0000 |
| LGBM vs LR | 6.7308 | 0.0095 | 0.5208 | 0.4705 | 6.2761 | 0.0122 |
| LGBM vs CatB | 0.9846 | 0.3211 | 0.0000 | 1.0000 | 0.9800 | 0.3222 |
| XGB vs RF | 55.5769 | 0.0000 | 18.8929 | 0.0000 | 36.4804 | 0.0000 |
| XGB vs LR | 14.4509 | 0.0001 | 0.8372 | 0.3602 | 14.2231 | 0.0002 |
| XGB vs CatB | 6.9605 | 0.0083 | 0.0556 | 0.8137 | 7.6034 | 0.0058 |
| RF vs LR | 6.3867 | 0.0115 | 6.9189 | 0.0085 | 2.0069 | 0.1566 |
| RF vs CatB | 37.9694 | 0.0000 | 14.7000 | 0.0001 | 22.3676 | 0.0000 |
| LR vs CatB | 4.0479 | 0.0442 | 0.3556 | 0.5510 | 3.6148 | 0.0573 |
|  |  |  | **10 years** |  |  |  |
| LGBM vs XGB | 10.2979 | 0.0013 | 2.4000 | 0.1213 | 7.0312 | 0.0080 |
| LGBM vs RF | 39.9633 | 0.0000 | 9.0250 | 0.0027 | 30.6667 | 0.0000 |
| LGBM vs LR | 8.6646 | 0.0032 | 0.0816 | 0.7751 | 10.6055 | 0.0011 |
| LGBM vs CatB | 14.5833 | 0.0001 | 7.3143 | 0.0068 | 6.6122 | 0.0101 |
| XGB vs RF | 23.1125 | 0.0000 | 4.9655 | 0.0259 | 17.6471 | 0.0000 |
| XGB vs LR | 1.4962 | 0.2213 | 0.2250 | 0.6353 | 3.5604 | 0.0592 |
| XGB vs CatB | 2.4407 | 0.1182 | 3.3750 | 0.0662 | 0.1143 | 0.7353 |
| RF vs LR | 6.4793 | 0.0109 | 7.7576 | 0.0053 | 1.3750 | 0.2410 |
| RF vs CatB | 17.6471 | 0.0000 | 0.2667 | 0.6056 | 20.2500 | 0.0000 |
| LR vs CatB | 0.0078 | 0.9296 | 4.9706 | 0.0258 | 2.3936 | 0.1218 |

**Table S-3.** Comparison of calibration performance across predictive models.

|  | ΔBS (mean) | 95% CI (lower) | 95% CI (upper) | p-value |
| --- | --- | --- | --- | --- |
| Models | **all-cause mortality** | | | |
|  | | **1 year** |  |  |
| CatBoost vs LR | 0.002687 | 0.000382 | 0.004904 | 0.0218 |
| CatBoost vs RF | 0.000769 | -0.000254 | 0.001789 | 0.1444 |
| CatBoost vs LGBM | 0.000094 | -0.001145 | 0.001349 | 0.8924 |
| CatBoost vs XGB | 0.000014 | -0.001269 | 0.001261 | 0.9842 |
|  |  | **3 years** |  |  |
| LGBM vs LR | 0.006408 | 0.003144 | 0.009629 | 0.0002 |
| LGBM vs RF | 0.002262 | 0.000763 | 0.003704 | 0.0034 |
| LGBM vs CatBoost | 0.000648 | -0.000325 | 0.001631 | 0.2030 |
| LGBM vs XGB | 0.000400 | -0.000585 | 0.001384 | 0.4216 |
|  |  | **5 years** |  |  |
| LGBM vs LR | 0.004926 | 0.001513 | 0.008409 | 0.0054 |
| LGBM vs RF | 0.001126 | -0.001053 | 0.003299 | 0.3006 |
| LGBM vs CatBoost | -0.000020 | -0.001311 | 0.001281 | 0.9748 |
| LGBM vs XGB | -0.000062 | -0.000985 | 0.000868 | 0.9072 |
|  |  | **10 years** |  |  |
| XGB vs LR | 0.006982 | 0.003596 | 0.010499 | 0.0000 |
| XGB vs RF | 0.003141 | 0.001136 | 0.005085 | 0.0024 |
| XGB vs CatBoost | 0.001674 | 0.000501 | 0.002832 | 0.0048 |
| XGB vs LGBM | 0.001395 | 0.000545 | 0.002256 | 0.0024 |
| Breast cancer-specific | | | | |
|  |  | **1 year** |  |  |
| LR vs RF | -0.002869 | -0.005215 | -0.000414 | 0.0228 |
| LR vs CatBoost | 0.002462 | -0.000220 | 0.005313 | 0.0764 |
| LR vs LGBM | 0.005716 | 0.003124 | 0.008465 | 0.0000 |
| LR vc XGB | -0.001124 | -0.003557 | 0.001443 | 0.3718 |
|  |  | **3 years** |  |  |
| LGBM vs LR | 0.006883 | 0.003768 | 0.009972 | 0.0000 |
| LGBM vs RF | 0.001049 | -0.000470 | 0.002560 | 0.1834 |
| LGBM vs CatBoost | 0.000658 | -0.000299 | 0.001643 | 0.1748 |
| LGBM vs XGB | 0.000230 | -0.000733 | 0.001245 | 0.6574 |
|  |  | **5 years** |  |  |
| XGB vs LR | 0.004379 | 0.000963 | 0.007812 | 0.0140 |
| XGB vs RF | -0.000205 | -0.002373 | 0.001945 | 0.8664 |
| XGB vs CatBoost | 0.000023 | -0.001083 | 0.001119 | 0.9408 |
| XGB vs LGBM | 0.000149 | -0.000613 | 0.000907 | 0.6856 |
|  |  | **10 years** |  |  |
| CatBoost vs LR | 0.008436 | 0.005066 | 0.011804 | 0.0000 |
| CatBoost vs RF | 0.002691 | 0.001029 | 0.004280 | 0.0014 |
| CatBoost vs LGBM | 0.001789 | 0.000148 | 0.003432 | 0.0320 |
| CatBoost vs XGB | 0.001297 | 0.000280 | 0.002322 | 0.0110 |

**Note:** The table presents the mean difference in Brier Score (ΔBS) for each model, along with the corresponding 95% confidence interval (95% CI) and p-values derived from pairwise statistical tests.

(A)	(B)	(C)

(D)

(E)	(F)	(G)

(H)

**S.Fig. 3.** Precision-Recall curves comparing machine-learning models for mortality prediction in breast cancer patients. Plots A-D show results for all-cause mortality at 1, 3, 5, and 10 years, respectively, while plots E-H present the corresponding results for breast cancer-specific mortality at the same time horizons.

A - 1 Year (CatBoost)	B - 3 Year (LightGBM)	C - 5 Year (LightGBM)

D - 10 Year (XGBoost)	E - 1 Year (LghtGBM)	F - 3 Year (LightGBM)

G - 5 Year (XGBoost)	H - 10 Year (CatBoost)

**S.Fig. 4.** Receiver operating characteristic (ROC) curves illustrating the robustness of the model with the highest AUC-ROC under Gaussian noise injection applied to the continuous variable age. The solid line represents the original (unperturbed) model, while dashed lines correspond to increasing noise levels (1%, 5%, and 10% of the standard deviation of age), based on 500 Monte Carlo simulations. Curves represent mean ROC performance across simulations. Plots A-D show results for all-cause mortality at 1, 3, 5, and 10 years, respectively, while plots E-H present the corresponding results for breast cancer-specific mortality at the same time horizons. The diagonal dashed line indicates chance-level discrimination.

**Table S-4.** Hyperparameters of the machine learning models used in the study

| Model | Parameter | Tested values | Description |
| --- | --- | --- | --- |
| Logistic Regression | C | [0.01, 0.1, 1, 10, 100] | Inverse regularization parameter. |
|  | penalty | ['l1', 'l2'] | L1 (lasso) or L2 (ridge). |
|  | solver | ['liblinear'] | Optimization for L1 and L2. |
|  | max_iter | [100, 500] | Maximum number of iterations. |
| Random Forest | n_estimators | [100, 200, 500] | Number of trees in the forest. |
|  | max_depth | [4, 8, 12] | Maximum tree depth. |
|  | min_samples_leaf | [1, 5, 10] | Minimum number of samples required at a leaf node. |
|  | min_samples_split | [2, 5, 10, 30] | Minimum number of samples to split an internal node. |
|  | criterion | ['gini', 'entropy'] | Function used to measure the quality of the split. |
| XGBoost | n_estimators | [100, 300, 500] | Number of weak estimators. |
|  | learning_rate | [0.01, 0.05, 0.1] | Learning rate. |
|  | max_depth | [3, 6, 9, 12] | Maximum tree depth. |
|  | subsample | [0.7, 0.8, 0.9] | Proportion of samples used per tree. |
|  | colsample_bytree | [0.7, 0.8, 0.9] | Proportion of variables used in each tree. |
| CatBoost | iterations | [100, 200, 300, 500] | Number of iterations. |
|  | learning_rate | [0.05, 0.1, 0.2] | Learning rate. |
|  | max_depth | [3, 5, 7] | Maximum tree depth. |
|  | grow_policy | ['Depthwise', 'SymmetricTree'] | Tree growth strategy. |
|  | subsample | [0.6, 1.0, 2] |  |
| LightGBM | n_estimators | [200, 300, 500] | Number of weak estimators. |
|  | learning_rate | [0.05, 0.1, 0.2] | Learning rate. |
|  | max_depth | [3, 5, 7] | Maximum tree depth. |
|  | num_leaves | [20, 40] | Maximum number of leaves per tree. |
|  | colsample_bytree | [0.7, 0.9] | Proportion of variables used in each tree. |
